# *Enterococcus faecalis* GP1764 induces an early differential gene expression in the intestine on key pathways related to cellular immune response and gut barrier function in chickens

**DOI:** 10.1016/j.psj.2026.107604

**Published:** 2026-08-13

**Authors:** M. Hussain, T. Jové-Juncà, O. Aizpurua, N. Tous, E. Casals, J. Langa, N. París, B. Jimenez-Moya, E. Rodríguez-Gallego, A. Esteve-Codina, M. Ballester, A. Alberdi, M.H. Kogut, J. Tarradas

**Affiliations:** a IRTA, Animal Nutrition, Mas Bové, 43120, Constantí, Catalonia, Spain; b MoBioFood Research Group, Department of Biochemistry and Biotechnology, Universitat Rovira i Virgili, c/Marcel·lí Domingo n°1, 43007, Tarragona, Spain; c IRTA, Genetics and Animal Breeding, Torre Marimon, 08140, Caldes de Montbui, Catalonia, Spain; d Center for Evolutionary Hologenomics, Globe Institute, University of Copenhagen, Copenhagen, Denmark; e Centro Nacional de Análisis Genómico (CNAG), Baldiri Reixac 4, 08028, Barcelona, Spain; f Universitat de Barcelona (UB), Barcelona, Spain; g Southern Plains Agricultural Research Center, USDA-ARS, College Station, TX, USA

**Keywords:** *Enterococcus faecalis*, Innate-like T cells, Inflammation, Probiotics, Immunomodulation

## Abstract

The aim of the present study was to elucidate the mode of action of *Enterococcus faecalis* GP1764 in improving performance traits during the starter phase by analyzing genome-wide gene expression and its interaction with microbial populations in the intestine of chickens challenged with an NSP-rich diet. At day 7, microbiota populations from ileal and cecal contents and transcriptomics from jejunal and cecal mucosa were analyzed between Control **(Ctrl)** and *Enterococcus faecalis* GP1764 **(*****EntF)*** groups. Results from microbiota analysis demonstrated that *EntF* shifted β-diversity indices in ileum (neutral (*p=* 0.006) and phylogenetic (*p=* 0.006)) and caecum (phylogenetic (*p=* 0.017)). Transcriptomics revealed 43 differentially expressed genes for *EntF vs.* Ctrl in the jejunal mucosa. Of these, *MHCY-36* (MHC-I-Related), *RAG2* and *MUC19-like* genes were upregulated in *EntF vs.* Ctrl, protein-coding genes with immunomodulatory capacities as supported by GSEA and Cytoscape-ClueGo pathway analyses. Results suggest an intestinal immunomodulation induced through presentation of B vitamins metabolites, synthetized by *EntF*, to an undescribed subset of innate-like unconventional T lymphocytes in chickens, similar to MAIT cells in mammals. These cells could contribute to antibacterial responses and repair of damaged barrier tissue after inflammatory processes. The upregulation of the *MUC19-like* gene expression observed in the jejunal mucosa can protect gut integrity via the promotion of mucus production by goblet cells. Finally, *RAG2*, involved in V(D)J coding segments recombination in B- and T-cells may provide a greater recognition of foreign invaders, allowing the animals to efficiently fight against pathogenic infections. Collectively, these results suggest an important role of *EntF* in promoting the capacity of animals to rapidly act against pathogenic challenges, herein, inducing resilience towards dietary ingredients with anti-nutritional activity that impart moderate inflammation in chickens.

## Introduction

Poultry production is essential for meeting the growing global demand for animal protein, with chickens being the most widely farmed poultry species worldwide (FAO, 2023). The broiler chicken sector has made significant advances in the last decades to increase meat yield through genetics, breeding, and nutritional strategies (Elwinger et al., 2016; Vaziri et al., 2022). However, the massive use of antibiotics has led to the emergence of antimicrobial resistances (**AMR**) in poultry pathogens, posing a significant threat to public health (Tarradas et al., 2020; Tous et al., 2022). In response to the ban of antibiotics as growth promoters in the European Union, alternative products are being intensively studied, among which probiotics have emerged as promising candidates capable of delivering similar benefits (mainly reduction of intestinal inflammation and modulation of microbiota populations) to those of antibiotics (FAO, 2006; Gadde et al., 2017; Hashemi et al., 2016; Hemarajata and Versalovic, 2013; Shehata et al., 2020; Tarradas et al., 2020).

On the other hand, it is well known that diets rich in soluble non-starch polysaccharides (NSP) negatively affect nutrient digestibility and intestinal viscosity causing an increase of inflammatory processes in the digestive tract and, finally, reducing the performance in poultry (Choct et al., 2010; Bach Knudsen, 2014; Kim et al., 2022b). In fact, it has been demonstrated that probiotics can partially mitigate the NSP-induced disturbances in nutrient digestibility and absorption (Tous et al., 2022). In our previous studies, diets rich in soluble NSP without enzymes have effectively been used as a challenge model to study and characterize the immunomodulatory effects of probiotics in counteracting inflammatory processes in the intestine (Tarradas et al., 2020; Tous et al., 2022).

Different studies demonstrated that some dietary probiotics can modulate intestinal immune system, improve performance, and reduce the prevalence of pathogens in chickens (Al-Khalaifa et al., 2019; Wu et al., 2019). Furthermore, they can also enhance nutrient digestibility and influence intestinal microbiota composition (Majidi-Mosleh et al., 2017). The gut microbiome, in turn, plays a key role in various aspects of host health, including defense against foreign antigens, immune system maturation, maintenance and repair of the epithelial mucosa, and the provision of essential nutrients and vitamins (Lin Lan, 2017). Therefore, fostering and maintaining beneficial host–microbiota interactions is a crucial aspect within the “One Health” framework (Zinsstag et al., 2011). However, despite their widespread use in the poultry industry and the beneficial effects of these probiotics on chickens' gastrointestinal tract function and physiology, the molecular mechanisms by which probiotics modulate the host immune system remain largely unclear (Abd El-Hack et al., 2020).

Recent studies have highlighted the capacities of non-pathogenic *Enterococcus faecalis* strains in poultry, demonstrating its ability to improve nutrient absorption (Franz et al., 2011), enhance intestinal barrier function (Fu et al., 2022), inhibit pathogens infection and establishment (Ford et al., 2022; Jahić and Cerovac, 2022), and modify gut microbiota (Hussain et al., 2024). However, *E. faecalis* remain a subject of controversy due to its dual identity as both a commensal bacterium or a potential emerging antimicrobial-resistant (AMR) pathogen (Hammerum et al., 2010; O’Dea et al., 2019). According to a 2022 assessment by the European Food Safety Authority (EFSA), *E. faecalis* poses a particular challenge as an AMR threat due to its commensal nature and opportunistic behavior, which masks accurate estimations of its prevalence, incidence, and clinical impact in poultry (EFSA Panel on Animal Health and Welfare (AHAW), 2022). Similarly, in humans, Daca and Jarzembowski (Daca and Jarzembowski, 2024) reviewed the distinct influences on vitamins production, nutrients metabolism, and intestinal pH maintenance, considering *E. faecalis* as ‘‘Friend and Foe’’ simultaneously.

This nature necessitates the quest for integrating in-depth omics and functional analysis to evaluate the potential of specific strains of *E. faecalis* as probiotic candidates in poultry production systems. In this sense, transcriptomic approaches have been increasingly used to investigate host responses in poultry under different physiological, pathogenic, and environmental conditions. Direct functional insights provided by microbial genomics and genome-resolved metagenomics represent a significant advancement in the analysis of metabolic capacities of bacteria (Eisenhofer et al., 2024; Segura-Wang et al., 2021). Shotgun metagenomics enables the reconstruction of microbial genomes through metagenomic assembly and binning, yielding metagenome-assembled genomes (**MAGs**), which can be annotated to provide functional capabilities of each genome (Shaffer et al., 2020; Tyson et al., 2004). This strategy represents a powerful tool to characterize and predict functionalities of promising probiotic strains, able to decipher their modes of action when these functionalities are contrasted with host transcriptomics. However, to date, studies integrating host transcriptomics with performance and microbiota data in response to dietary probiotics that improve intestinal health and enhance productivity in poultry remain scarce.

The current study is part of a large project and extends the scope of a previous experiment, in which the administration of three probiotic strains, individually or as a blend, were tested during 35 days in two different *in vivo* trials (Hussain et al., 2024). In this research we observed that *Enterococcus faecalis* GP1764 (***EntF***) improved performance traits (BW, ADG, FCR, and EPEF) and altered intestinal microbiota composition, particularly during the starter phase, in chickens fed a challenging diet rich in NSPs. The improvement on the performance at 8 d showed that this probiotic could partially counteract the antinutritional effects induced by the presence of NSPs in the diet. Moreover, the shift observed in microbiota populations at 7 d suggested that the first week of life may be an open window to modify microbiota populations with direct implications on performance at the end of the growing period, as previously proposed by (Dal Pont et al., 2021). However, the mode of action of *EntF* on intestinal microbiota and on the host’s intestinal epithelium remains unknown.

The aim of the present study was to elucidate the mode of action of *Enterococcus faecalis* GP1764, through genome-wide gene expression analysis of the intestinal mucosa, to decipher possible changes in the chickens’ mucosal transcriptome during the starter phase, as well as the interaction with the microbial populations. This will contribute to the understanding of how the probiotic is able to improve the growth performance of chickens through the epithelial response and microbiota modulation during the first days of life.

## Materials and methods

### Ethics statement

The design and execution of this study followed the national—Spanish Royal Decree 53/2013—and international—European Directive 2010/63/EU—legislation regarding animal experimentation for scientific purposes. The experimental procedures were approved by the Ethics Committee of the Generalitat de Catalunya, Spain (Project number 11697).

### In vivo experimental design and diets

This work is part of larger study previously published. In present research article, only the data and samples from the starter phase and from the **Ctrl** and ***EntF*** (at 5·10^7^ CFU/bird/d) obtained in the Trial 1 were considered (Hussain et al., 2024). Briefly, animals were randomly assigned to 6 pens with 36 birds/pen per treatment. Wheat/maize, soybean and rye-based basal diets were formulated to meet or exceed FEDNA recommendations (FEDNA, 2018). Diets were formulated to induce a moderate inflammation in the upper digestive tract through increasing the percentage of soluble NSPs and without adding exogenous enzymes (Hussain et al., 2024). The negative control was the group without probiotic. A control group without challenge (basal diet + exogenous enzymes) was not included in this study given that the Performance Objectives published by Aviagen give information about the performance to be reached at the end of the study (BW of 2441 g at 35d) in optimal conditions (Aviagen, 2022).

The dose of probiotic to be used in the present study (*E. faecalis* GP1764 at 5·10^7^ CFU/bird/d) was selected based on previous studies with similar design (Alizadeh et al. (2024); Giannenas et al., 2012; Kim et al., 2024). The probiotic-feed mixture was prepared at a ratio of 1:3 and administered in 4-6 g of feed per bird and day. Feed and water were provided *ad libitum* consumption.

On 7 d, 2 birds per pen (n = 12 per treatment and day) were euthanized and sampled. These chicks were randomly selected and marked at 0 d to avoid observer biases in subsequent samplings. Mucosa from two fragments of 3.0 × 1.0 cm of jejunum and cecum tissues were scraped and collected for transcriptomics analyses. Approximately 3 g of jejunal and cecal contents were collected for microbiota analysis. All samples were immediately frozen in liquid nitrogen and preserved at -80°C until analysis.

### RNA-Seq analysis

#### RNA extraction sequencing

Total RNA from jejunal and cecal mucosa was extracted using the Chemagic™ RNA Tissue 10 mg Kit H96 (ref. CMG-1212; Revvity, Baesweiler, Germany) following the manufacturer's instructions. Total RNA was quantified using the Qubit® RNA BR Assay Kit (Thermo Fisher Scientific Q10210, Eugene, OR), and RNA integrity was assessed with the RNA 6000 Nano Kit on the Bioanalyzer 2100 system (Agilent Technologies, USA). RNA-Seq libraries were prepared using the KAPA Stranded mRNA-Seq Kit for Illumina platforms (Illumina, Inc., USA), following the manufacturer's instructions. Briefly, 500 ng of total RNA was used for poly-A mRNA enrichment, and the resulting blunt-ended double-stranded cDNA was 3′-adenylated. Illumina-compatible adapters with unique dual indexes and unique molecular identifiers (Integrated DNA Technologies) were ligated to the cDNA, and the ligation products were enriched by 15 cycles of PCR. Final libraries were quantified using the DNA 7500 Assay on the Bioanalyzer 2100 (Agilent Technologies, USA). Sequencing was performed on an Illumina NovaSeq 6000 system (Illumina, Inc., USA) in paired-end mode with a read length of 2 × 51 bp, according to the manufacturer’s protocol for dual indexing. Image analysis, base calling, and quality scoring were carried out using Real Time Analysis software version 3.4.4, followed by the generation of FASTQ files.

#### Bioinformatics analysis of RNA-seq

RNA-seq reads were mapped against the *Gallus gallus* reference genome GRCgb7 with STAR 2.7.10a (Dobin et al., 2013) using ENCODE parameters. Genes were quantified with RSEM 1.3.1 (Li and Dewey, 2011) using the Ensembl 112 annotation. Rarely expressed genes were filtered out by only retaining those with at least one count-per-million reads (CPM) in at least six samples to reduce noise and improve the reliability and statistical power of the differential expression analysis. This procedure ensures a minimal and consistent expression level across samples. Genes with very low counts contribute little biological information and are more likely to reflect noise or technical variability. Thus, this filtering step primarily has a statistical impact, reducing the number of tests, improving model fitting, and enhancing the robustness of downstream differential expression analyses.

Differential gene expression between *EntF* and Ctrl groups were performed with limma R package v3.54.2 (Ritchie et al., 2015), using TMM normalization. The voom function (Law et al., 2014) was used to transform the count data into log_2_-counts per million (**logCPM**), estimate mean-variance relationship and to compute observation-level weights. Considering that none of the genes passed the filter of FDR<5%, to eliminate the dependency on the gene selection criteria, and assuming that phenotypic differences are manifested by small but consistent changes in a group of genes, a Gene set enrichment analysis (**GSEA**) was performed with the pre-ranked list of genes by the limma t moderated statistic, using FGSEA 1.24.0 and Reactome.db R packages (Korotkevich et al., 2016). The list of genes included in the pathway “Adaptive Immune System” detected by GSEA was analysed through the ClueGO v2.5.9 plug-in of Cytoscape v3.9.1 (Load Marker List: *Gallus gallus*; Gene Ontology “Immune System Process EBI-UniProt_GOA-ACAP-ARAP”; updated 13/03/2025″; GO Tree interval: 4 to 12; GO Term/Pathway selection: 3 genes/cluster and < 4% genes (Bindea et al., 2009)) to further study which specific pathways were involved.

In parallel, the EdgeR R package (Robinson et al., 2010) was also used to identify differentially expressed (**DE**) genes between *EntF* and Ctrl groups. After correcting for multiple testing, genes with a Fold Change (**FC**) between groups greater than 1.2 (i.e. |log_2_FC| > 0.26) and an FDR < 0.1 were classified as DE.

### Microbiota analysis

The procedure carried out for the DNA extraction, 16S amplicon sequencing, and data analysis was fully described in Hussain et al. (2024) (Hussain et al., 2024). Briefly, DNA was extracted from cecal and ileal content samples by using DNeasy 96 PowerSoil Pro Kit (Qiagen, Germany), following the manufacturer's instructions. Transcribed 16S rRNA libraries targeting V1–V3 region from bacterial population were sequenced by utilizing MiSeq Illumina sequencing platform (Illumina, 2 × 300 bp kit, San Diego, CA, USA). For the bacterial libraries the 27F (5′-AGRGTTTGATCMTGGCTCAG–3′) / 519R (5′-GTNTTACNGCGGCKGCTG-3′) primer set was used. DNA reads were compiled in FASTq files.

Amplicon sequencing reads were demultiplexed based on library indices using AdapterRemoval (Schubert et al., 2016). Primer locations were identified Using Cutadapt 1.18 (Martin, 2011), and the reads were reverse-complemented. Taxonomic assignment was done by the naive Bayesian classifier method as implemented in DADA2 (Callahan et al., 2016) in R 4.4.2 (Dessau and Pipper, 2008), against SILVA 16S rRNA database (version 138.2).

For the microbiota assessment, diversity analyses and visualization were carried out using Hill numbers as implemented in the R package *hilldiv2* (Alberdi and Gilbert, 2019).

### Genomic characterization of enterococcus faecalis

Multiple replicates of *E. fecalis* isolated from the original probiotic mixture were resuspended for genomic characterization. DNA extraction was carried out using the in-house developed procedure for recovery of bacterial DNA, standardized in the Earth Hologenome Initiative (Leonard and Alberdi, 2024; Pietroni et al., 2025). DNA was sheared through ultrasonication to 500 bp-long fragments, and short-read sequencing libraries were generated using the BEST protocol (Carøe et al., 2018). Sequencing was carried out in an Illumina HiSeq2000 platform (San Diego, CA, USA).

After quality filtering and adaptor trimming using fastp, individual assemblies were generated using metaSpades before binning contigs into MAGs using an ensemble approach relying on Maxbin2, Metabat2 and CONCOCT, followed by Metawrap refinement. This approach was selected over direct genome assembly to minimize the impact of contaminants in the reconstruction of the draft genome of *E. faecalis*.

Genome quality was assessed using Checkm2, taxonomic annotation was conducted using GTDB-tk, and functional annotation was carried out using DRAM. Subsequently, raw gene annotations were distilled into Genome-Inferred Functional Traits (**GIFTs**) using DistillR (available at https://github.com/anttonalberdi/distillR), which employs a curated database comprising 328 metabolic pathways and modules from KEGG (Kanehisa et al., 2017) and Metacyc (Karp et al., 2002) databases.

### Statistical analysis

For microbiota analyses, alpha diversity comparisons were conducted using the Wilcoxon rank-sum test. Group-wise dispersion homogeneity was evaluated using the betadisper function from the vegan R package (Oksanen, 2015). Differences in community composition were assessed through PERMANOVA, implemented via the adonis function in vegan. Microbial community variation was represented using Nonmetric Multidimensional Scaling (NMDS) ordination plots, constructed from distance matrices with the vegan and ggplot2 packages (Wickham, 2007). Differential abundance analysis of microbiome count data was carried out using the Analysis of Compositions of Microbiomes with Bias Correction 2 (ANCOM-BC2) method (Lin and Peddada, 2020).

For transcriptomic analyses, differential gene expression was performed with limma R package v3.54.2 (Ritchie et al., 2015), using TMM normalization and transformed to logCPM through the voom function (Law et al., 2014). The GSEA was performed with the pre-ranked list of genes by the limma t moderated statistic, using FGSEA 1.24.0 and Reactome.db R packages (Korotkevich et al., 2016). EdgeR R package (Robinson et al., 2010) was also used to identify DE genes. Genes with a FC greater than 1.2 (i.e. |log_2_FC| > 0.26) and FDR < 0.1 were classified as DE.

For intestinal microbiota and transcriptomics analyses, the experimental unit was the animal.

## Results

### Probiotic effects on cecal and ileal microbiota diversity and enrichment at day 7

**Fig. 1:**
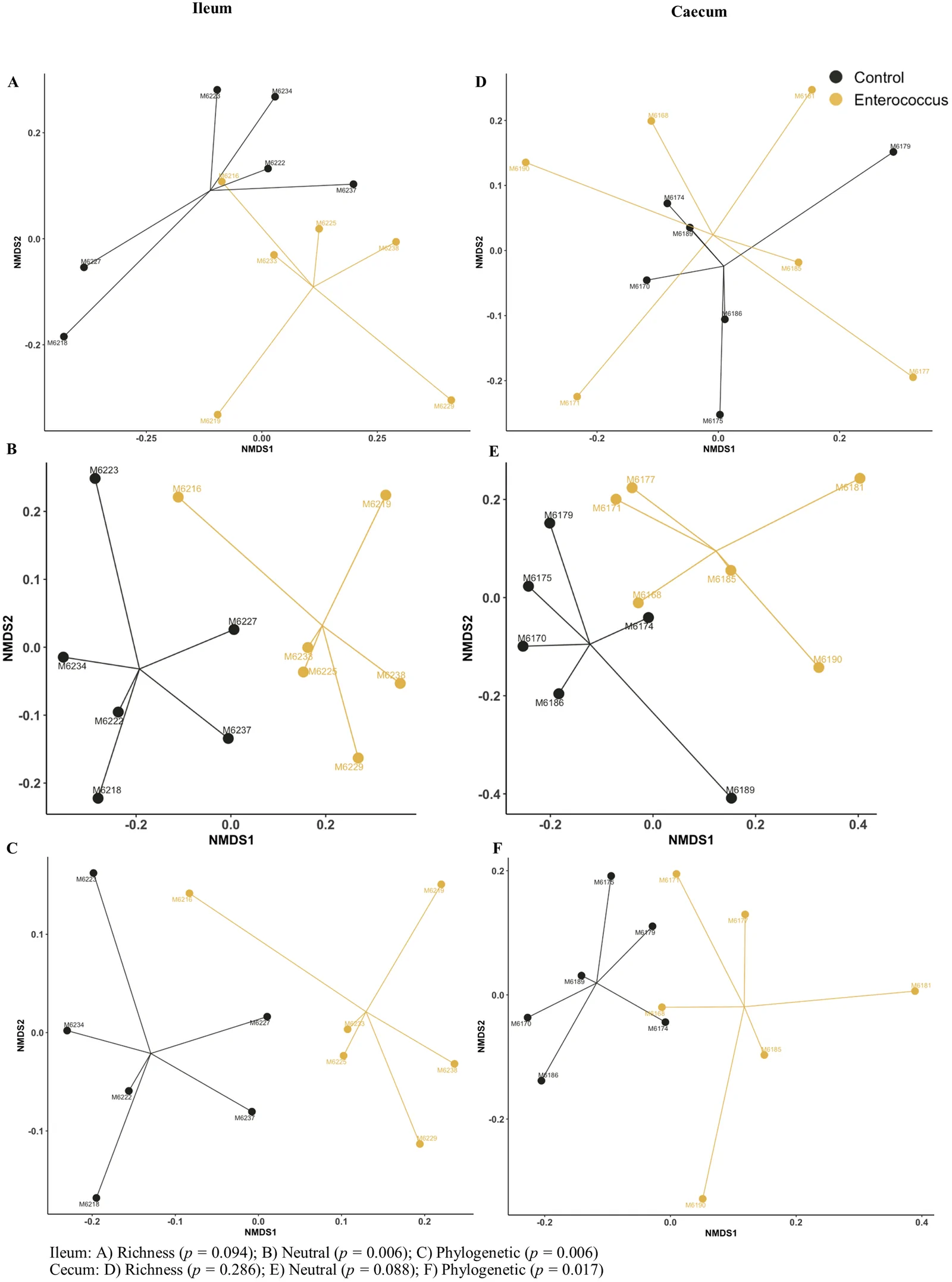
Effects of *EntF* administration on ileal and cecal β**-**diversity at 7d.

No significant differences in α-diversity were observed in either the caecum or ileum in terms of richness, neutral or phylogenetic indices (Table 1). In the ileum, neutral and phylogenetic β-diversity indices were significantly different compared to the control group (neutral: *p* = 0.006, R2= 0.404, Fig. 1B; phylogenetic: *p* = 0.006, R2 = 0.402, Fig. 1C). Furthermore, a trend toward significance was observed in the richness index (*p* = 0.094, R2= 0.113) (Fig. 1A). In the cecum, only the phylogenetic index was significantly different in the *EntF*-fed group compared to the control (*p* = 0.017, R2 = 0.134) (Fig. 1F), while there was a trend in neutral index for the same comparison (*p* = 0.088, R2 = 0.122) (Fig. 1E).

**Table 1:** Effects of *EntF* on cecal and ileal microbiota α-diversity at 7d.

	Ctrl	*EntF*	*SD*	*P-value*
Caecum				
Richness	146.5	145.5	18.36	*0.810*
Neutral	45.3	43.2	7.95	*0.940*
Phylogenetic	24.4	26.3	3.93	*0.820*
Ileum				
Richness	57.17	52.67	13.40	*0.820*
Neutral	7.38	7.26	2.30	*0.700*
Phylogenetic	3.72	3.56	0.70	*0.390*

Values are presented as least squares means. *SD:* Standard deviation. a-b Values within a column without a common superscript differ P < 0.05.

**Fig. 2:**
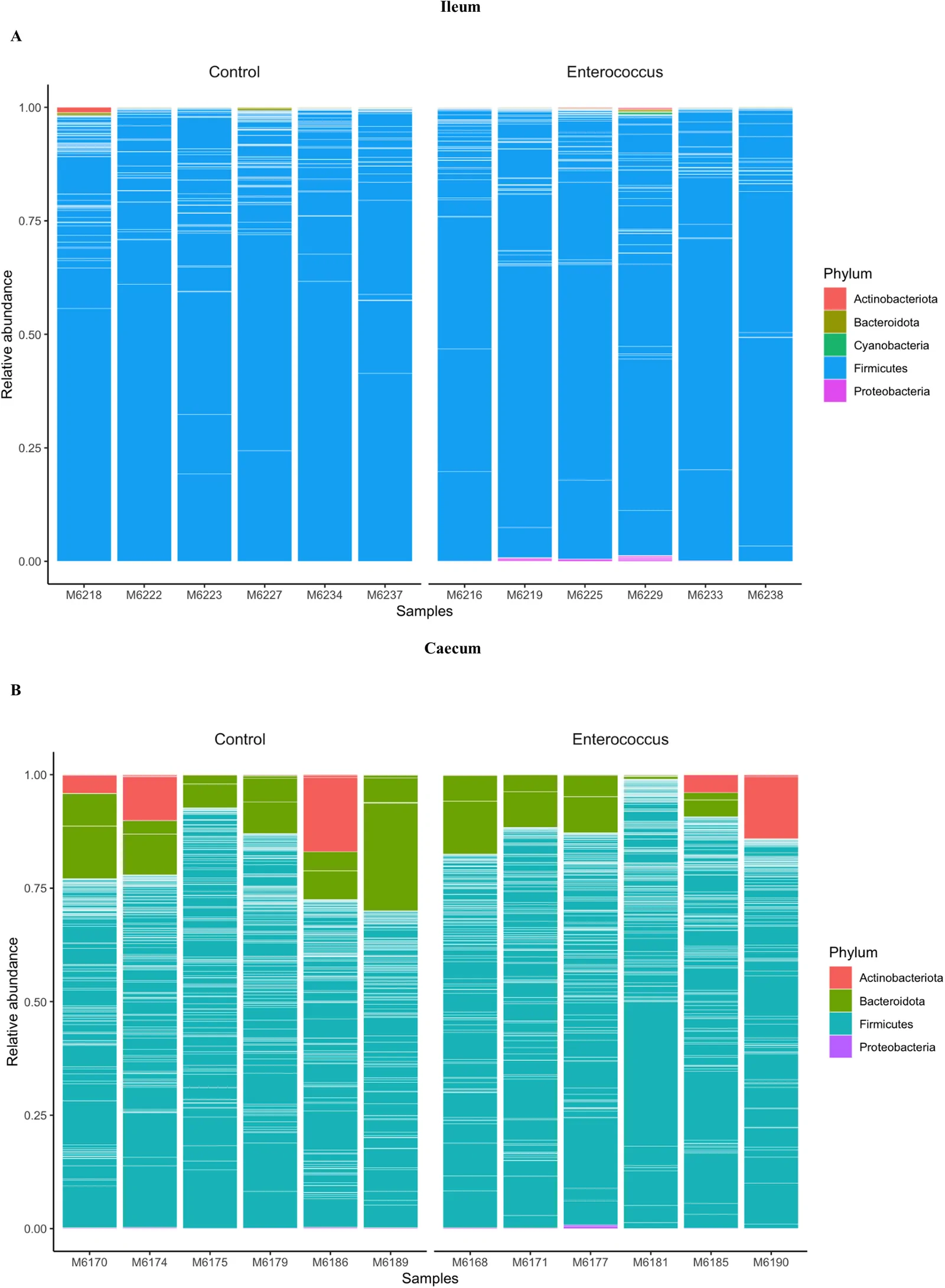
Effects of *EntF* administration on taxonomic classification of ileal and cecal microbiota at d 7. Stacked bar chart of intestinal bacteria by relative abundance at phylum level.

In the ileum, over 99% of the ASVs belonged to the phylum Firmicutes (Control = 99.43 ± 0.65, *EntF* = 99.05 ± 1.02) (Fig. 2A). In the caecum, Firmicutes remained the dominant phylum in both groups (Ctrl = 79.33 ± 8.78; *EntF* = 88.71 ± 5.74), followed by Bacteroidota (Ctrl = 15.22 ± 8.12; *EntF* = 8 ± 6.97), Actinobacteriota (Ctrl = 5.26 ± 6.94; *EntF* = 3.07 ± 5.62) and Proteobacteriota (Control = 0.19 ± 0.1; *EntF* = 0.22 ± 0.3) (Fig. 2B). No significant differences in the phylum-level abundance were observed between groups (ANCOM-BC2: *p* > 0.05).

In the ileum, ASV-level ANCOM-BC2 analysis detected 81 ASVs unique to the control group and 69 unique to the *EntF* group. Among the 10 ASVs that were differentially abundant, two *Lactobacillus* were enriched in *EntF* group, while eight (five *Lactobacillus, Negativibacillus, Erysipelatoclostridium* and *Blautia*) were more abundant in the control group (Fig. 3A). In the caecum, 105 ASVs were exclusive to the Ctrl group and 139 to *EntF* group. A total of 26 ASVs were differentially abundant, with 7 enriched in the control group, and 19 in the *EntF* group (Fig. 3B). In both groups, all of the enriched ASVs belonged to Firmicutes, most of them from the family Lachnospiraceae (55.56%). However, the top three enriched ASVs in *Ent* group belonged to *Lactobacillus, Butyricicoccus* and unidentified genera from the Bacillaceae family. An ASV identified *Enterococcus* was almost significant in *EntF* group (*p* = 0.057) in caecum.

**Fig. 3A:**
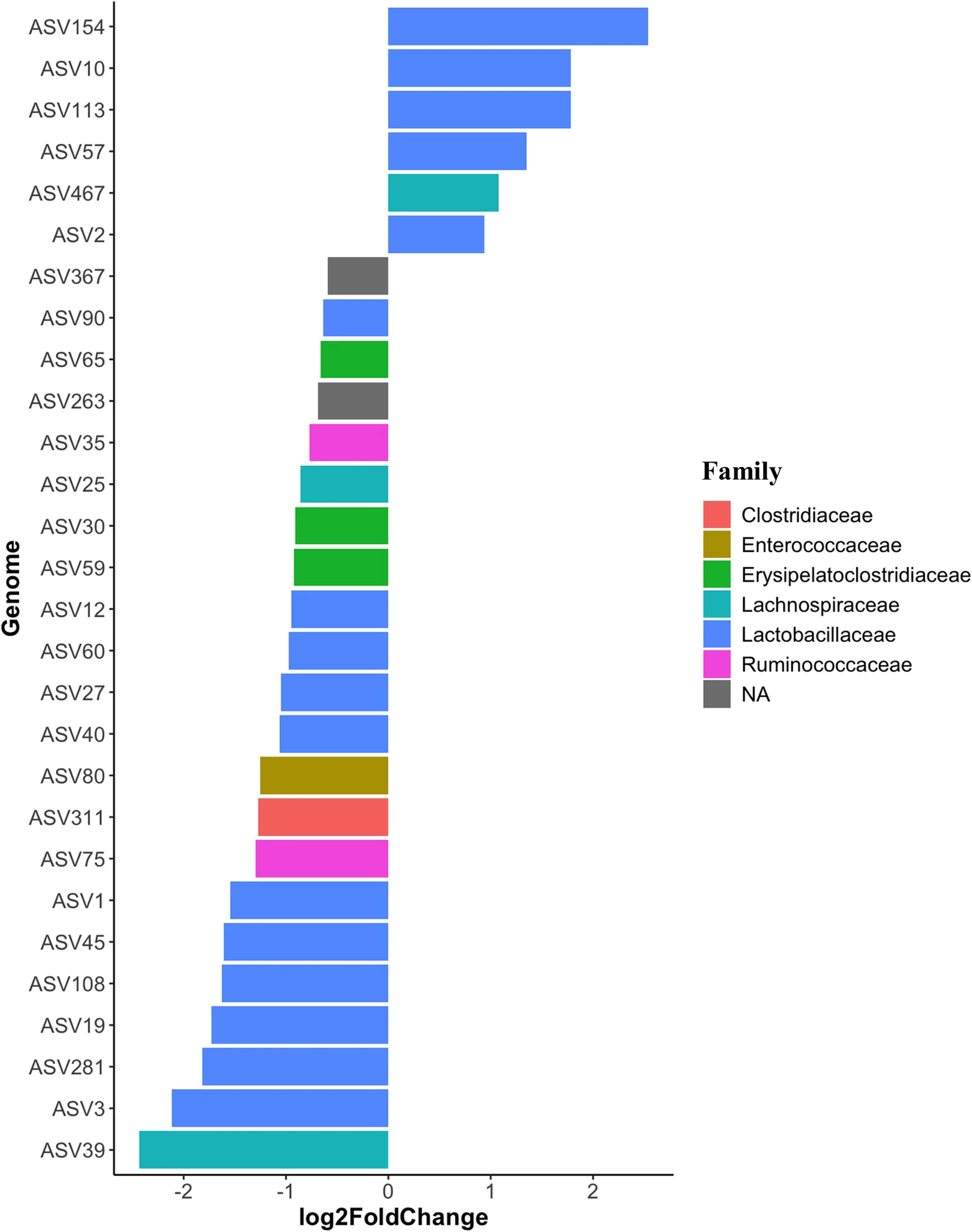
Enrichment analysis Ancombc2 at 7 d in ileum (*EntF* vs Ctrl) family (*p* < 0.1)**.**

**Fig. 3B:**
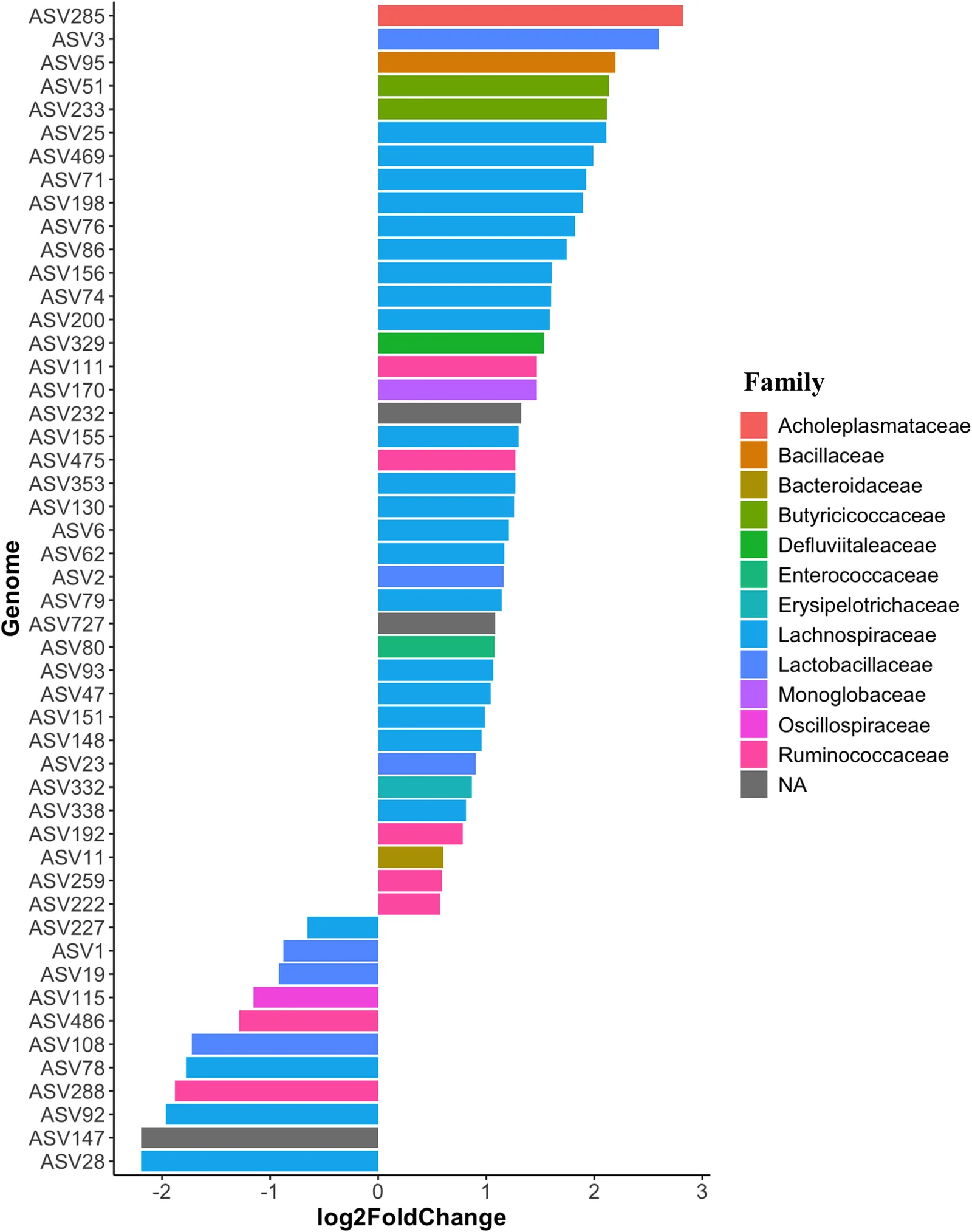
Enrichment analysis Ancombc2 at 7 d in cecum (*EntF* vs Ctrl) family (*p* < 0.1)**.**

Differential abundance analysis at the genus level revealed distinct microbial signatures between the *EntF* and control chicken groups. Ten genera—*Intestinibacter, CHKCI001, Hydrogenoanaerobacterium, Fournierella, Lachnospira, UCG-009, Candidatus Soleaferrea, Gordonibacter*, and unidentified genera belonging to the families Butyrococcaceae and Erysipelotrichaceae—were exclusive to the *EntF* group. In contrast, seven genera—*UC5-1-2E3, Anaerofustis, CAG-352, 28-4, Proteus, Klebsiella*, and an unidentified member of *Lactobacillales*—were unique to the control group.

Additionally, eight ASVs showed significant differential abundance, all of which were detected only in the *EntF* group. These included ASVs classified as *Eisenbergiella, Monoglobus, CAG-56, Intestinimonas*, and *Paludicola* and unidentified genera belonging to *Lachnospiraceae* and *Bacillaceae*.

### Gene set enrichment analysis and functional analysis of core genes detected

The GSEA analysis detected different upregulated key pathways associated with the adaptive immune system and endoplasmic reticulum (**ER**) to Golgi apparatus transport, while pathways related to the cell cycle were downregulated in *EntF* compared to Ctrl at d 7 in jejunum (Table 2). Contrarily, no pathways identified in the caecum showed significant differences between groups at d 7 (Table S1).

**Table 2:** Gene set enrichment analysis (GSEA) at 7d in jejunal mucosa (*EntF vs* Ctrl).

Pathway	NES	Padj	Size
Membrane_Trafficking	1.99	<0.001	151
Vesicle−mediated_transport	1.90	<0.001	158
Cargo_concentration_in_the_ER	2.00	0.010	8
Asparagine_N−linked_glycosylation	1.85	0.012	66
Transport_of_small_molecules	1.64	0.016	145
Adaptive_Immune_System	1.64	0.019	141
Transport_to_the_Golgi_and_subsequent_modification	1.84	0.021	46
Rab_regulation_of_trafficking	1.94	0.021	24
ER_to_Golgi_Anterograde_Transport	1.84	0.022	42
Intra−Golgi_traffic	1.91	0.034	16
Intra−Golgi_and_retrograde_Golgi−to−ER_traffic	1.75	0.034	53
RAB_GEFs_exchange_GTP_for_GDP_on_RABs	1.89	0.036	18
Retrograde_transport_at_the_Trans−Golgi−Network	1.88	0.042	15
GTP_hydrolysis_and_joining_of_the_60S_ribosomal_subunit	-3.21	<0.001	35
SRP−dependent_cotranslational_protein_targeting_to_membrane	-3.20	<0.001	37
Formation_of_a_pool_of_free_40S_subunits	-3.14	<0.001	40
Nonsense_Mediated_Decay_(NMD)_independent_of_the_Exon_Junction_Complex	-3.12	<0.001	38
Translation	-3.07	<0.001	70
Cap−dependent_Translation_Initiation	-3.06	<0.001	44
Eukaryotic_Translation_Initiation	-3.06	<0.001	44
Nonsense_Mediated_Decay_(NMD)_enhanced_by_the_Exon_Junction_Complex	-2.80	<0.001	43
Cell_Cycle_Checkpoints	-2.58	<0.001	54
Formation_of_the_ternary_complex,_and_subsequently,_the_43S_complex	-2.55	<0.001	23
Ribosomal_scanning_and_start_codon_recognition	-2.55	<0.001	23
Activation_of_the_mRNA_upon_binding_of_the_cap−binding_complex and_eIFs,_and_subsequent_binding_to_43S	-2.55	<0.001	23
Translation_initiation_complex_formation	-2.55	<0.001	23
Cell_Cycle	-2.43	<0.001	140
Cell_Cycle,_Mitotic	-2.39	<0.001	130
Activation_of_ATR_in_response_to_replication_stress	-2.35	<0.001	12
Synthesis_of_DNA	-2.33	<0.001	5
Mitotic_Spindle_Checkpoint	-2.30	<0.001	2
Metabolism_of_RNA	-2.19	<0.001	131

*NES*; Normalized Enrichment Score quantifying the degree to which a gene set is over-represented at the top or bottom of a ranked gene list.

**Fig. 4:**
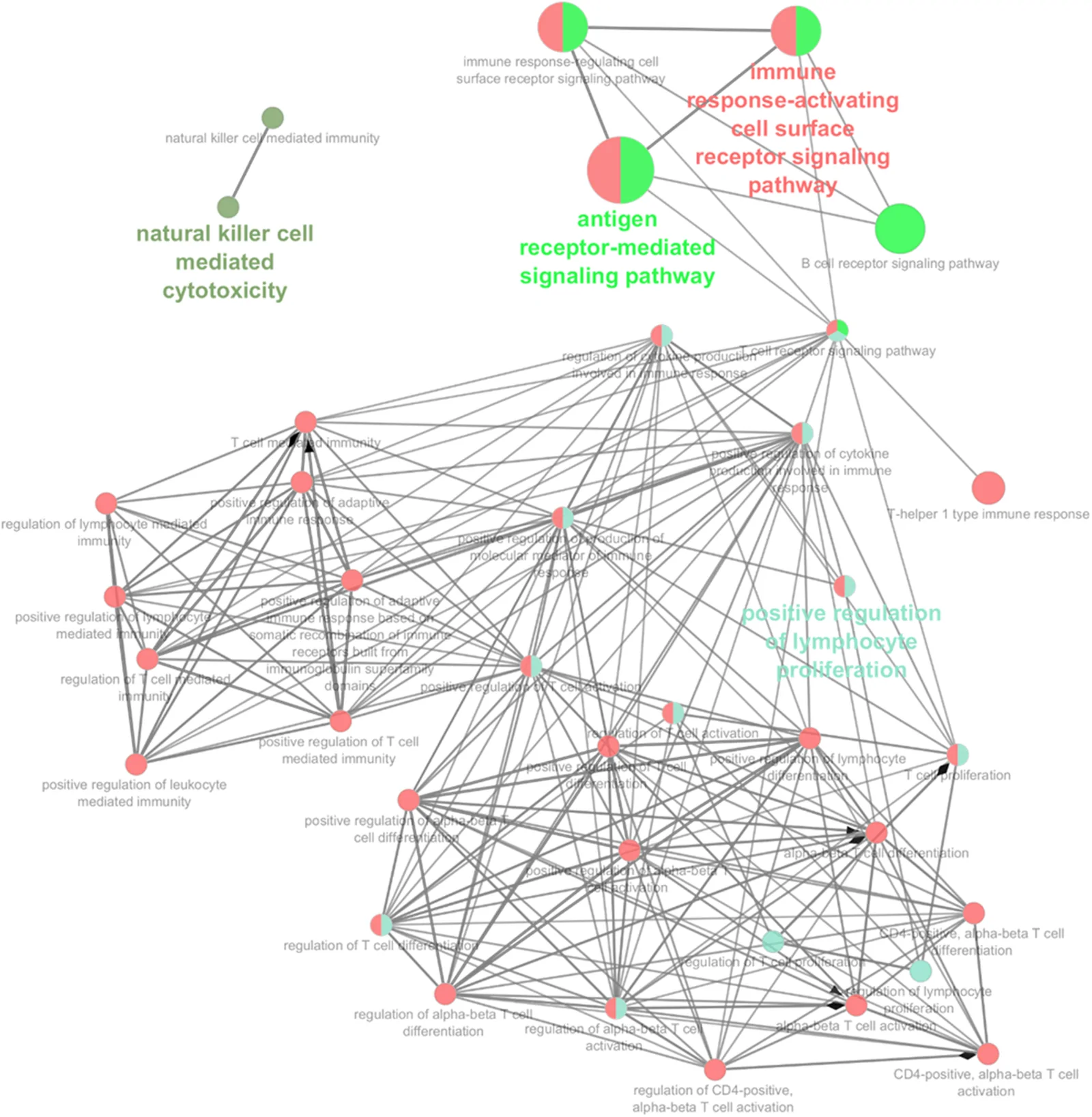
Schematic network of nodes and edges for Adaptive immune system pathway by Cluego – Cytoscape identified in GSEA analysis.

In order to characterize the immunomodulatory effects of *EntF* on the host transcriptome, a functional annotation for genes identified in GSEA analysis was done for the key pathway “Adaptive Immune System” at 7 d in jejunum. Fig. 4 and Table S3 show the functional terms identified, and their interconnections (237 edges and 37 nodes as representative terms and pathways) observed in ClueGo - Cytoscape for the 75 genes (Table S2) identified in GSEA analysis. The results showed four functional categories with 51 functional terms in total (Fig. 4): “Immune-response-activating cell surface receptor signaling pathway (62,75%), Positive regulation of lymphocyte proliferation (23.53%), “Antigen receptor-mediated signaling pathway (9.8%)” and “Natural killer cell mediated cytotoxicity (3.92%) (Table S3). The GO terms “Immune response-activating cell surface receptor signaling pathway”; “immune response-regulating cell surface receptor signaling pathway”; and “antigen receptor-mediated signaling pathway”, involved in the regulation and activation of adaptive immune responses, showed the highest number of associated genes (*CBLB, GRB2, HRAS, NFATC2, PCASP1, PRKCB, SOS1, SYK, TRAF6*). The next most enriched GO term was “regulation of T cell activation” with eight related genes (*CBLB, KLHL22, KLHL25, PCASP1, PTPN11, SOS1, SYK, TRAF6*) (Table S2).

### Differentially expressed genes between *EntF* vs Ctrl groups identified by EdgeR

The differential expression analysis from jejunal and cecal mucosa expression data was also analyzed using EdgeR methodology to explore DE genes in *EntF* vs Ctrl groups at 7d. Results are presented in Table 3, Table 4, respectively. A total of 37 DEs were detected in jejunum at 7 d of which 9 were upregulated and 28 were downregulated in *EntF* vs Ctrl groups (Table 3). Among the top regulated immune-related genes, *MHCY36* (FC= 85.63), *RAG2* (FC = 47.18) and *MUC19-like* (FC = 12.34) were upregulated while *CLDN18* (FC^-1^ = 268.73) was downregulated in *EntF* fed animals compared to control ones.

**Table 3:** GEA - EdgeR analysis. Differentially expressed genes at 7d in jejunal mucosa (*EntF vs* Ctrl).

Transcript ID (Ensembl)	Gene name*	Protein name (or recommended name)	Log_2_FC	*p-value*	FDR
ENSGALG00010003311	Unknown	Uncharacterized protein	9.57	4.08·10^-5^	0.047
ENSGALG00010002303	MHCY36	Ig-like domain-containing protein / *MHCY36* gene	6.42	3.45·10^-5^	0.047
ENSGALG00010006062	Unknown	-	4.78	4.36·10^-5^	0.047
ENSGALG00010018624	LOC112531388	CCHC-type domain-containing protein	3.90	2.58·10^-5^	0.040
ENSGALG00010018616	LOC770705	Integrase	1.31	4.20·10^-5^	0.047
ENSGALG00010025239	RAG2	V(D)J recombination-activating protein 2	5.56	2.25·10^-4^	0.098
ENSGALG00010011173	Unknown	Uncharacterized protein - MUC19 Like	3.62	8.70·10^-5^	0.074
ENSGALG00010019253	HBAD	Hemoglobin alpha, subunit D	1.16	2.16·10^-4^	0.098
ENSGALG00010008265	LOC396098	Ig-like domain-containing protein – *Bu-1* gene	1.07	1.97·10^-4^	0.095
ENSGALG00010007954	PSCA	Prostate stem cell antigen	-10.36	4.53·10^-5^	0.047
ENSGALG00010023364	CTRB1	Chymotrypsinogen B1	-10.18	6.62·10^-6^	0.019
ENSGALG00010011020	SFTPA2	Surfactant protein A2	-9.65	3.01·10^-6^	0.014
ENSGALG00010011009	SFTPA1	Surfactant protein A1	-9.36	1.12·10^-6^	0.007
ENSGALG00010029156	Unknown	Uncharacterized protein	-9.15	7.17·10^-6^	0.019
ENSGALG00010025507	ATP12A	Sodium/potassium-transporting ATPase subunit alpha	-8.92	1.02·10^-5^	0.021
ENSGALG00010029944	LOC427816	Fibrinogen C-terminal domain-containing protein	-6.60	3.99·10^-5^	0.047
ENSGALG00010014952	C21orf58	Chromosome 21 open reading frame 58	-5.64	6.83·10^-6^	0.019
ENSGALG00010014696	SPINK5	Serine peptidase inhibitor, Kazal type 5	-5.29	1.77·10^-5^	0.032
ENSGALG00010000137	LOC425940	Saposin B-type domain-containing protein	-5.23	8.01·10^-6^	0.019
ENSGALG00010004785	NKX2-8	NK2 homeobox 8	-8.94	9.45·10^-5^	0.077
ENSGALG00010020133	Unknown	Uncharacterized protein	-8.80	1.27·10^-4^	0.081
ENSGALG00010004654	NKX2-1	NK2 homeobox 1	-8.55	1.40·10^-4^	0.081
ENSGALG00010025594	UPK1B	Uroplakin 1B	-8.26	1.55·10^-4^	0.085
ENSGALG00010022713	PNLIPRP1	Pancreatic lipase related protein 1	-8.11	7.05·10^-5^	0.066
ENSGALG00010024572	CLDN18	Claudin 18	-8.07	1.64·10^-4^	0.088
ENSGALG00010007908	IRX1	Iroquois homeobox 1	-7.99	1.74·10^-4^	0.090
ENSGALG00010021208	CCDC33	Coiled-coil domain-containing protein 33	-7.89	6.10·10^-5^	0.060
ENSGALG00010020939	CTRC	Chymotrypsin C	-7.68	1.07·10^-4^	0.078
ENSGALG00010023541	PRSS2	Protease, serine 1	-6.29	1.25·10^-4^	0.081
ENSGALG00010006574	CPA1	Carboxypeptidase A1	-6.25	2.10·10^-4^	0.098
ENSGALG00010011790	AMY2A	Amylase, alpha 2A (pancreatic)	-6.18	1.26·10^-4^	0.081
ENSGALG00010017555	CELA2A	Chymotrypsin like elastase family member 2A	-5.80	1.08·10^-4^	0.078
ENSGALG00010026706	KRT24	Keratin 24	-4.92	1.93·10^-4^	0.095
ENSGALG00010028460	CRLF1	Cytokine receptor like factor 1	-4.87	1.78·10^-4^	0.090
ENSGALG00010024137	Unknown	Uncharacterized protein	-4.87	1.04·10^-4^	0.078
ENSGALG00010017806	CELA3B	Peptidase S1 domain-containing protein	-4.54	1.30·10^-4^	0.081
ENSGALG00010013928	CCNO	Cyclin O	-4.24	2.26·10^-4^	0.098

⁎ Name obtained using Database *Gallus gallus* or Orthologues (Ensembl, BioMart tool), UniProtKB annotation, and/or Protein Blast (NCBI). Last accession 2025, 23rd May. Log_2_FC: Log_2_ Fold Change; FDR: False Discovery Rate.

**Table 4:** GEA - EdgeR analysis. Differentially expressed genes at 7d in cecal mucosa (*EntF vs* Ctrl).

Transcript ID (Ensembl)	Gene name*	Protein name (or recommended name)	Log_2_FC	*p-value*	FDR
ENSGALG00010018170	SLC5A11	Solute carrier family 5-member 11	6.48	2.46·10^-08^	<0.001
ENSGALG00010008307	MEP1A	Meprin A subunit	6.04	1.09·10^-06^	0.004
ENSGALG00010010900	SLC34A2	Solute carrier family 34-member 2	5.83	6.63·10^-07^	0.003
ENSGALG00010023983	SUSD2	Sushi domain containing 2	5.65	8.11·10^-08^	<0.001
ENSGALG00010001385	MME	Membrane metalloendopeptidase	4.08	3.58·10^-06^	0.011
ENSGALG00010017681	ALPI	Alkaline phosphatase	2.61	1.63·10^-05^	0.039
ENSGALG00010024773	APOA1	Apolipoprotein AI	1.47	7.71·10^-06^	0.021
ENSGALG00010003258	Unknown	Uncharacterized protein	10.42	2.58·10^-05^	0.053
ENSGALG00010010295	Actin	Actin, alpha skeletal muscle	4.75	3.88·10^-05^	0.053
ENSGALG00010011448	AvBD10	Avian beta-defensin 10	3.54	7.88·10^-05^	0.100
ENSGALG00010011173	Unknown	Uncharacterized protein	3.40	3.29·10^-05^	0.053
ENSGALG00010023369	ACE	Angiotensin I converting enzyme	1.95	2.99·10^-05^	0.053
ENSGALG00010019253	HBAD	Hemoglobin alpha, subunit D	1.49	3.82·10^-05^	0.053
ENSGALG00010008048	LY6EL	UPAR/Ly6 domain-containing protein	-2.44	3.43·10^-05^	0.053

⁎ Name obtained using Database *Gallus gallus* or Orthologues (Ensembl, BioMart tool), UniProtKB annotation, and/or Protein Blast (NCBI). Last accession 2025, 23rd May. Log_2_FC: Log_2_ Fold Change; FDR: False Discovery Rate.

In caecum at 7d, 14 DEs were detected including 13 upregulated and only one downregulated gene in *EntF* group compared to Ctrl group (Table 4). *ALPI* (FC = 6.10) and *AvBD10* (FC = 11.63) genes were upregulated.

### Genome-inferred functional traits (GIFT)

**Fig. 5:**
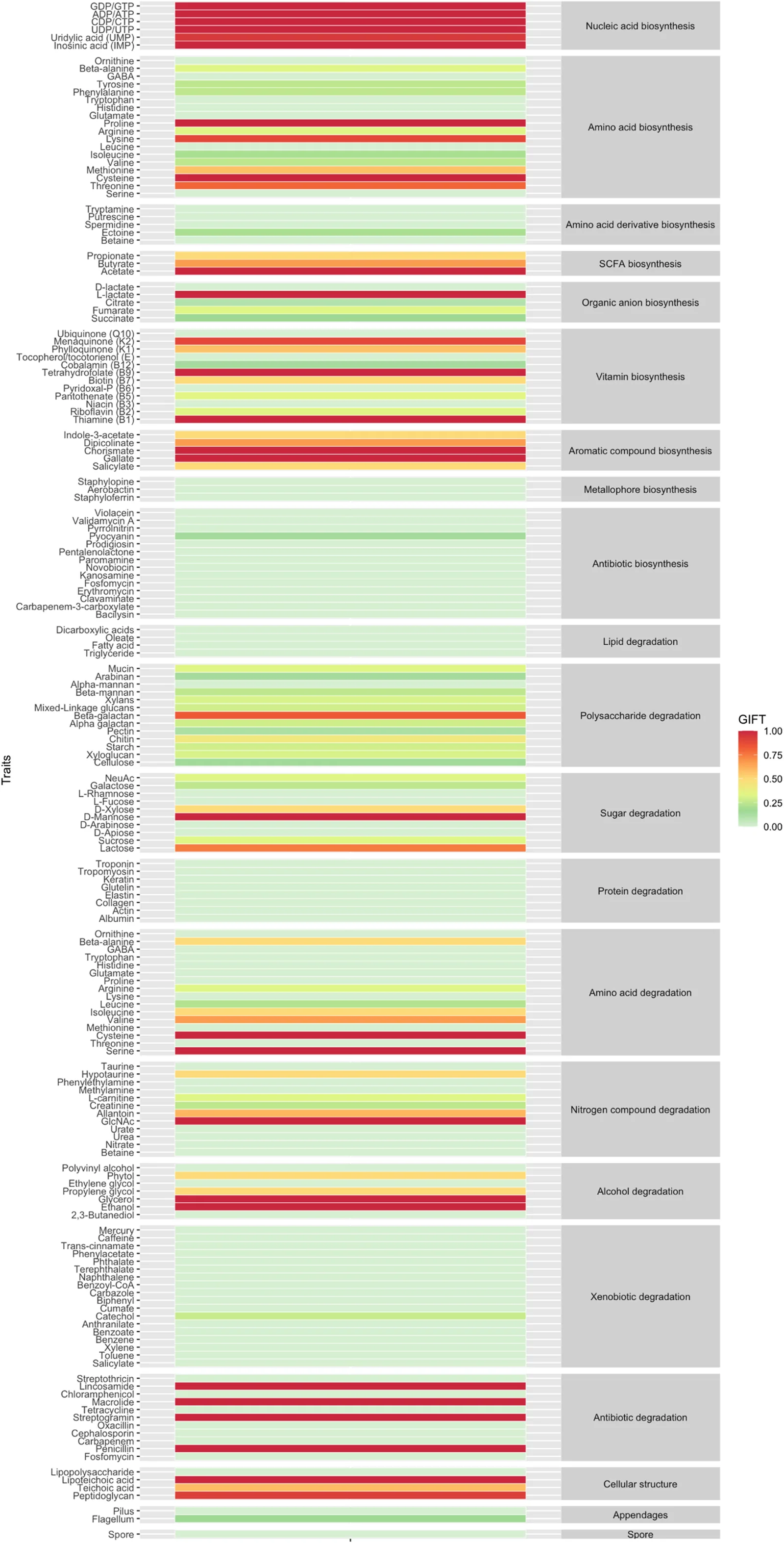
Genome inferred functional traits (GIFT) analysis of *Enterococcus faecalis* GP1764.

The draft genome of *EntF* was reconstructed at an estimated completion of 99.98%, with 2.43% predicted contamination. Taxonomic annotation validated the expected taxonomy, with 98.77% similarity against the closest *Enterococcus faecalis* present at the GTDB database. Gene prediction yielded a list of 3030 genes, out of which 2103 were functionally annotated against the KEGG metabolic database, 554 were annotated against the virulence factor database, and 139 were annotated against the carbohydrate active enzyme database. The distillation of gene annotations into genome-inferred functional traits yielded a functional profile of *EntF* with relevant capacities on amino acid, short-chain fatty acids (**SCFA**), vitamins, and aromatic compounds biosynthesis. Moreover, functions on polysaccharide, sugar, amino acid, nitrogen, alcohol and antibiotic degradation were detected (Fig. 5).

## Discussion

In the present study, we evaluated the effects of *E. faecalis* GP1764 (*EntF*) probiotic*,* on gene expression profiles in gut, employing transcriptomics, and on microbiota populations to understand the improvements on performance observed during the first week of age in broiler chickens challenged with a diet rich in NSP (Hussain et al., 2024). Our previous findings demonstrated that supplementation with the *EntF*-based probiotic significantly improved broiler performance during the starter phase (0–8 days), as evidenced by increased BW (3.4%), ADG (4.5%), EPEF (7.6%), alongside a reduction in FCR (-3.3%) (Hussain et al., 2024).

*Enterococcus faecalis* has been shown to modulate gut microbiota composition and increase beneficial bacterial counts, such as *Lactobacillus*, in the chicken cecum (Hussain et al., 2024; Shehata et al., 2020). Similar effects have been observed in other species, where *E. faecalis* supplementation resulted in distinct microbiota profiles (Wang et al., 2024). Current research shows that the improvements in performance occurred without significant changes in α-diversity indices, suggesting that overall microbial richness and evenness remained stable between the *EntF*-treated and control groups. However, the observed shifts in β-diversity, indicate substantial changes in microbial community composition and structure. While the probiotic treatment explained a greater proportion of microbial variation in the ileum (40%) than in the caecum (13%), higher number of differentially abundant ASVs were identified detected in the cecum. This pattern may reflect intrinsic differences in microbial community structure along the intestinal tract. The cecum harbors a more diverse and complex microbial community, which may provide a larger pool of taxa capable of responding to perturbations, thereby increasing the likelihood of detecting differentially abundant ASVs. In contrast, microbial biomass and diversity are much lower in the ileum, resulting in a less complex community that may be more susceptible to compositional shifts, which could explain the stronger influence of *EntF* on β-diversity in this region.

Firmicutes remained the dominant phylum in both intestinal sections, consistent with early-life poultry microbiota (Burrows et al., 2025). Nevertheless, *EntF* supplementation induced finer-scale shifts at the ASV and genus levels, particularly within Firmicutes, which may contribute to the improved performance outcomes. In the ileum, differentially abundant ASVs were primarily from the *Lactobacillaceae* family. In contrast, the caecum exhibited a greater number of unique and enriched ASVs in the *EntF* group, especially from the *Lachnospiraceae* family, which are known for their capacity to ferment non-digestible carbohydrates into SCFAs, which support gut barrier function, and reduce inflammation (Polansky et al., 2015). Although the genera with the highest log-fold change in *EntF* group in the differential analysis did not belong to *Lachnospiraceae*, are bacteria that have been previously linked with benefit in chicken’s gut health (e.g., *Lactobacillus, Butyricicoccus*, and unidentified *Bacillaceae* members) (Eeckhaut et al., 2016; Yue et al., 2024). The fact that several ASVs from the same genera such as *Lactobacillus, Blautia*, and *Anaerostipes* were enriched in both groups, suggests selective promotion of different strains within shared core genera. When performing the same analysis after aggregating at the genus level, enriched genera were found only in the *EntF* group. The presence of enriched ASVs from genera such as *Hydrogenoanaerobacterium*, has also been associated with increased BW and ADG (Zhang et al., 2021a). Increases in *Fournierella* were also linked to immune development (Liu et al., 2023). These evidences further supports the performance-enhancing effects of *EntF*. Additionally, several genera uniquely present in the control group, including *Proteus* and *Klebsiella*, are known opportunistic pathogens associated with dysbiosis and reduced growth (Veloo et al., 2022). Their absence in the *EntF* group may reflect a protective effect conferred by the probiotic. However, further research is needed to clarify the mechanistic pathways by which *E. faecalis* influences gut ecology and promotes host development.

Aiming to understand how the administration of *EntF* can modulate the host’s metabolic and immune pathways, genome-inferred metabolic trait analysis was conducted on *EntF* bacterial genome to determine its capacity to degrade or produce key biomolecules. Our results show that *EntF* contains genes involved in metabolic functions known to improve or maintain intestinal health and performance. These include synthesis of SCFA and vitamins, and degradation of polysaccharides, among other pathways with direct effects on intestinal microbiota and host digestive and immune systems.

On the other hand, transcriptomic analysis using EdgeR identified *MHCY36, RAG2*, and *MUC19-like* genes that are upregulated by *EntF* in jejunum, all with known immunomodulatory functions. Complementary analysis using GSEA revealed two important general pathways positively modulated, Adaptive_Immune_System and ER-to-Golgi apparatus transport. In this line, Cytoscape-ClueGo pathway analysis also showed an increase in the expression of genes belonging to pathways related to T-cell response in animals consuming probiotic *EntF*. The GO terms “Immune-response-activating cell surface receptor signaling pathway”, “Immune response-regulating cell surface receptor signaling pathway”; and “antigen receptor-mediated signaling pathway”, all involved in the regulation and activation of adaptive immune response, showed the highest number of associated genes. This pattern suggests a strong immunomodulatory capacity of *EntF* to direct T‑cell responses in the jejunal mucosa of broilers at 7 d, particularly through the surface receptor signaling pathways that govern their activation.

The differentially expressed genes identified in jejunal mucosa through the EdgeR analysis further supported this potential mode of action for *EntF.* Specifically, an increase in the expression of the major histocompatibility complex class I (MHC-I)-related gene was observed in *EntF-*fed animals. A novel Ig-like domain-containing protein (ENSGALG00010002303) was identified, showing 98.7% homology with MHC-Y class I heavy chain 36 – *MHCY36* gene (NP_001383095.1; Protein Blast - *Gallus gallus*, NCBI) and 40% homology with the human MHC class I-related gene protein – *MR1* gene (XP_011507765.1; Protein Blast – *Homo sapiens*, NCBI). This upregulation of *MHCY36* by *EntF* suggests a potential modulation of key T-cell recognition system during early life, which may influence the development of chicken’s cellular immune response. It is generally accepted that MHC-Y class I molecules do not present peptides to immune system due to their narrow binding groove to accommodate peptides (Goto et al., 2022; Hee et al., 2010), but recent mass spectrometry analysis have identified lysophospholipids as candidate ligands for MHC-Y class I (Gugiu et al., 2023). Similarly, *MR1* presents a range of small molecule antigens such as water-soluble vitamins derived from microbial metabolism to mucosal-associated invariant T (MAIT) cells (Ito and Yamasaki, 2024; McWilliam and Villadangos, 2024). These cells are a subset of innate-like unconventional T lymphocytes with a potent antimicrobial activity, and important roles in tissue homeostasis (Gao and Zhao, 2024), concomitantly expressing cytotoxicity and tissue-repair genes (Germain et al., 2024). Although MAIT cells are thought to play a protective role in gut, their dysregulation may lead to chronic inflammation (Fang et al., 2025). However, the precise mechanisms by which these cells balance protective immunity and inflammation in the gut remain poorly understood.

In humans, *MR1* ligands comprise various vitamin B2 (riboflavin)-derived metabolites that activate MAIT cells, as well as vitamin B9-related compounds that do not elicit these cells (McWilliam and Villadangos, 2018). Although the vitamin B9-derived metabolite 6-formylpterin (6-FP) potently antagonizes the presentation of bacteria-derived antigens to MAIT cells, it may have an immune-activation function through the engagement of yet-to-characterized non-classical MAIT cells or atypical MR1-T cells (Tang et al., 2022). Given the capacity of *EntF* to synthetize vitamin B9 (tetrahydrofolate), and perhaps vitamin B2 (riboflavin), we hypothesize that vitamins B9 and B2 synthesized by *EntF* could be presented through MHCY36 modulating the activation of an unknown and specific subset of ‘innate-like’ T-cells in chickens with similar effects to those of MAIT cells in mammals.

In parallel, our results demonstrate an increased expression of *RAG2* gene (ENSGALG00010025239) in jejunal mucosa, an essential component of the (RAG1-RAG2)_2_ recombinase (RAG) enzymatic complex. RAG recognizes specific recombination signal sequences flanking the 3′ end of the V, D, and J coding segments in immunoglobulin- and T cell receptor- genes and initiates the V(D)J recombination in both B and T cells (Kuo and Schlissel, 2009). This observation supports the hypothesis that *EntF* promotes T cell expansion, potentially enabling recognition of different antigens presented through MHC-Y complex. However, although the chicken α/β/γ/δ TCR repertoire has recently been characterized (Früh et al., 2024; Zhang et al., 2021b), the identification of the ligands remains poorly understood. To date, no T-cell subset population capable of detecting microbiota-derived B-vitamins has been described, however our results suggest its existence in poultry.

Additionally, our results suggest that *EntF* strain is also capable of strengthening innate immune defense in jejunal mucosa through promoting the expression of *MUC19-like* gene [(ENSGALG00010011173) with a homology of 58.8% with mucin-19-like gene (XP_061216547.1); Protein Blast – *Neopsephotus bourkii*, NCBI)], which in humans encodes coding for a gel-forming secreted mucin (Lang et al., 2006). Although the structural components and functional properties of mucins have been extensively studied in humans, little is known in poultry. The *MUC19* gene has not been annotated yet in the chicken reference genome and only five MUC genes have been described to date (reviewed in (Duangnumsawang et al., 2021b)). Considering the evidence obtained from transcriptomics, *EntF* could induce the expression of an undescribed mucin in the jejunum, and potentially via non-constitutive pathway of mucins secretion activated by external stimuli causing the exocytosis of goblet cells (Kim and Khan, 2013). However, the present results do not allow us to decipher the external stimuli capable of inducing the synthesis and excretion of this mucin.

The complexity of mucin carbohydrate structures synthesis is regulated by glycosylation within the Golgi apparatus of the goblet cells (Duangnumsawang et al., 2021a) followed by a mucin dimerization in the ER (Lang et al., 2006). This could explain the high number of activated pathways detected by GSEA related to Golgi apparatus-ER transport in jejunum. An alternative explanation could be the high metabolic activity of dendritic cells to present antigens to non-activated effector T cells. The increase in the synthesis, transport and secretion of MHC complex and accessory molecules is dependent on the expansion of the ER and Golgi by lipogenesis and synthesis of additional membranes (Manoury et al., 2022). Moreover, the different functions of dendritic cells, including antigen uptake and presentation, pose unique demands on the cellular organelles, including ER (Manoury et al., 2022).

Claudins are tight junction membrane proteins that are expressed in epithelia and endothelia and form paracellular barriers and pores that determine tight junction permeability. Notably, an important downregulation was observed in claudin-18 (*CLDN18*) gene expression in the jejunum of animals fed *EntF* treatment compared to Ctrl group. In chickens, little is known about the claudin-18 expression and its role in tight junction formation and regulation of paracellular transport between epithelial cells, but studies in other species observed that it is present in lung, stomach, duodenum and jejunum (Günzel and Yu, 2013). Although no differences were observed on the intestinal morphology (evaluated in (Hussain et al., 2024)) or on the necrotic enteritidis score (data not shown), it is well known that high amounts of dietary NSPs induce gut inflammation promoting sub-clinical alterations in intestinal integrity (Kogut et al., 2025). Therefore, it can be speculated that Ctrl animals overexpressed *CLDN18* gene to repair and maintain the intestinal epithelium structure and functionality, damaged by the presence of NSPs. In contrast, the administration of *EntF* would avoid these sub-clinical alterations caused by NSPs on the gut barrier epithelium. Therefore, the overexpression of CLDN18 in intestinal tissue could be used as a biomarker to assess both disease progression and tissue repair. To evaluate this hypothesis, further analyses, such as intestinal epithelial permeability assays, would be necessary. However, the absence of fresh samples precludes these analyses; consequently, validation of this assumption should be pursued in future experiments.

Our results also demonstrate a clear upregulated expression of important genes related to homeostasis maintenance (*ALPI*) and antimicrobial activity (*AvBD10*) in cecum mucosa at 7 days of age. The *ALPI* gene is reported to be involved in regulating gut microbiome composition and epithelial cell differentiation (Yang et al., 2015), LPS detoxification and protection of gut barrier integrity (Singh and Lin, 2021) and to reduce the activity of Toll-like receptor 4 agonist (Parlato et al., 2018). The functions of avian β-defensins include antibacterial activity, chemotaxis and activation of immune cells, the modulation of inflammation, and the maintenance of homeostasis through the activation of mucin synthesis (Yang et al., 2021). Moreover, the avian β-defensin-10 is an antimicrobial peptide with demonstrated antimicrobial efficacy against relevant pathogens (Munoz et al., 2023). Considering these functionalities, the administration of *EntF* could also have a direct effect on the chicken’s cecum promoting epithelium protection and increasing its innate antimicrobial capacity.

Finally, it is important to mention that other relevant genes were differently expressed between Ctrl and *EntF* treatments (Table 3, Table 4). However, based on current state of knowledge, a direct relationship between their differential expression and the observed improvement in birds’ performance cannot be clearly established. Therefore, further targeted analyses are required to better understand the effects on the animal.

The present study is focused on deciphering the immunomodulatory capacities of *EntF* on the host. Although several pathways have been identified as possible modes of action more research is needed to verify the differentially expressed genes by sequencing through qPCR analysis, and to understand others mode of action of *EntF* related to these genes’ expression and its implication on performance and health improvement.

## Conclusions

Current results indicate that the supplementation of broilers with the probiotic *EntF* during the first days of life was able to partially counteract a dietary challenge with NSP, promote intestinal barrier protection, gut health, and nutrient absorption and finally explain improvements on performance. The upregulation of *MHCY36* gene expression in jejunal mucosa, molecules responsible for the presentation of vitamin B metabolites (synthesized by *E. faecalis*), may be able to activate an undescribed subset of innate-like T-cells with antimicrobial activity and capacity to drive the cellular immune response towards a tissue homeostasis and repairment. These findings may also be attributed to substantial changes in intestinal microbial community composition and structure, but the link between microbiota changes and the differently expressed genes in the host is not yet fully understood. Future research is needed to validate these initial hypotheses.

## Accession information for sequencing

For total bacterial populations, data from MiSeq NGS assessment was submitted to the Sequence Read Archive (SRA) of the National Center for Biotechnology Information (NCBI) with the BioProject ID: PRJNA1126084. Data from Transcriptome Sequencing (RNA-seq) and WGS of *Enterococcus faecalis* GP-1764 were submitted to the SRA of NCBI with the BioProject ID: PRJNA1286969.

## CRediT authorship contribution statement

**M. Hussain:** Writing – review & editing, Writing – original draft, Visualization, Validation, Methodology, Investigation, Formal analysis, Data curation, Conceptualization. **T. Jové-Juncà:** Visualization, Software, Formal analysis. **O. Aizpurua:** Writing – original draft, Visualization, Software, Methodology, Formal analysis, Data curation. **N. Tous:** Writing – review & editing, Writing – original draft, Investigation, Formal analysis. **E. Casals:** Software, Formal analysis, Data curation. **J. Langa:** Formal analysis, Data curation. **N. París:** Resources, Methodology, Data curation. **B. Jimenez-Moya:** Resources, Methodology, Formal analysis. **E. Rodríguez-Gallego:** Writing – review & editing, Supervision, Investigation, Funding acquisition. **A. Esteve-Codina:** Writing – review & editing, Visualization, Software, Investigation, Formal analysis, Data curation. **M. Ballester:** Writing – review & editing, Writing – original draft, Software, Investigation, Formal analysis, Data curation. **A. Alberdi:** Writing – review & editing, Writing – original draft, Software, Methodology, Investigation, Formal analysis, Data curation. **M.H. Kogut:** Writing – review & editing, Validation, Supervision, Methodology, Investigation, Conceptualization. **J. Tarradas:** Writing – review & editing, Writing – original draft, Validation, Supervision, Resources, Project administration, Methodology, Investigation, Funding acquisition, Formal analysis, Data curation, Conceptualization.

## Disclosures

The authors: Muzahir Hussain, Teodor Jové-Juncà, Ostaizka Aizpurua, Núria Tous, Eloi Casals, Jorge Langa, Núria París, Beatriz Jiménez-Moya, Esther Rodríguez-Gallego, Anna Esteve-Codina, Maria Ballester, Antton Alberdi, Michael H. Kogut and Joan Tarradas reports no conflict of interests.

## Footnotes

Supplementary material associated with this article can be found, in the online version, at doi:10.1016/j.psj.2026.107604.
