# Supplementary material for: Enterococcus faecalis GP1764 induces an early differential gene expression in the intestine on key pathways related to cellular immune response and gut barrier function in chickens
Source: Poult Sci. 2026 Aug 13;105(12):107604. doi: 10.1016/j.psj.2026.107604 (PMC13594961; doi:10.1016/j.psj.2026.107604)
Supplement: Supplementary file 1 [file mmc1.docx]

EARLY-PROBIOTIC IMMUNOMODULATION IN BROILERS

***Enterococcus faecalis* GP-1764 induces an early differential gene expression in the intestine on key pathways related to cellular immune response and gut barrier function in chickens**

**M. Hussain*^ǁ^, T. Jové-Juncà^§^, O. Aizpurua^†^, N. Tous*, J. Langa^†^, N. París*, B. Jimenez-Moya*, E. Rodríguez-Gallego^ǁ^, A. Esteve-Codina^#^, M. Ballester^§^, A. Alberdi^†^, M.H. Kogut ^¶^, J. Tarradas***

*****IRTA, Animal Nutrition, Mas Bové, 43120 Constantí, Catalonia, Spain.

**^§^**IRTA, Genetics and Animal Breeding, Torre Marimon, 08140 Caldes de Montbui, Catalonia, Spain.

**^†^**Center for Evolutionary Hologenomics, Globe Institute, University of Copenhagen, Copenhagen, Denmark.

**^ǁ^**MoBioFood Research Group, Department of Biochemistry and Biotechnology, Universitat Rovira i Virgili, c/Marcel·lí Domingo n°1, 43007 Tarragona, Spain.

**^#^**Centre Nacional d’Anàlisi Genòmica (CNAG), Barcelona, Spain.

**^¶^**Southern Plains Agricultural Research Center, USDA-ARS, College Station, TX, USA.

Corresponding author: [joan.tarradas@irta.cat](mailto:joan.tarradas@irta.cat)

**Table S1:** Gene Set Enrichment Analysis (GSEA) at 7d in cecal mucosa (*EntF* vs Ctrl)

| Pathway | NES | *Padj* |
| --- | --- | --- |
| G_beta:gamma_signalling_through_PLC_beta | 1.77 | NS |
| Presynaptic_function_of_Kainate_receptors | 1.77 | NS |
| Visual_phototransduction | 1.76 | NS |
| Cooperation_of_PDCL_(PhLP1)_and_TRiC/CCT_in_G−protein_beta_folding | 1.75 | NS |
| Surfactant_metabolism | 1.73 | NS |
| G−protein_activation | 1.72 | NS |
| Acetylcholine_regulates_insulin_secretion | 1.71 | NS |
| Synthesis_of_IP3_and_IP4_in_the_cytosol | 1.71 | NS |
| G_alpha_(s)_signalling_events | 1.70 | NS |
| Metabolism_of_fat−soluble_vitamins | 1.68 | NS |
| Thrombin_signalling_through_proteinase_activated_receptors_(PARs) | 1.68 | NS |
| Retinoid_metabolism_and_transport | 1.67 | NS |
| Incretin_synthesis,_secretion,_and_inactivation | 1.65 | NS |
| Synthesis,_secretion,_and_inactivation_of_Glucagon−like_Peptide-1_(GLP-1) | 1.65 | NS |
| Free_fatty_acids_regulate_insulin_secretion | 1.56 | NS |
| Fatty_Acids_bound_to_GPR40_(FFAR1)_regulate_insulin_secretion | 1.56 | NS |
| Na+/Cl−_dependent_neurotransmitter_transporters | 1.54 | NS |
| G_alpha_(i)_signalling_events | 1.51 | NS |
| Chylomicron_assembly | 1.33 | NS |
| Synthesis,_secretion,_and_inactivation_of_Glucose−dependent_Insulinotropic_Polypeptide | 1.33 | NS |
| PDH_complex_synthesizes_acetyl−CoA_from_PYR | -1.91 | NS |
| Miscellaneous_transport_and_binding_events | -1.88 | NS |
| Golgi_Associated_Vesicle_Biogenesis | -1.83 | NS |
| UCH_proteinases | -1.81 | NS |
| Sulfur_amino_acid_metabolism | -1.81 | NS |
| MTOR_signalling | -1.80 | NS |
| Sensory_perception_of_salty_taste | -1.80 | NS |
| Neddylation | -1.79 | NS |
| Pyruvate_metabolism | -1.77 | NS |
| Signaling_by_Retinoic_Acid | -1.77 | NS |
| TP53_Regulates_Metabolic_Genes | -1.73 | NS |
| Metabolism | -1.73 | NS |
| Transport_of_connexons_to_the_plasma_membrane | -1.72 | NS |
| Autophagy | -1.64 | NS |
| Macroautophagy | -1.60 | NS |
| Endosomal_Sorting_Complex_Required_For_Transport_(ESCRT) | -1.51 | NS |
| Deubiquitination | -1.48 | NS |
| Deadenylation−dependent_mRNA_decay | -1.33 | NS |
| Receptor−type_tyrosine−protein_phosphatases | -1.33 | NS |
| Membrane_Trafficking | -1.31 | NS |

**Table S2:** List of genes obtained (GSEA) for “adaptive immune system pathway” in jejunum at 7d

| Gene stable Ensembl ID | Gene name (*Homo sapiens*) | Gene name (*Gallus gallus*) |
| --- | --- | --- |
| [ENSGALG00010000784](http://www.ensembl.org/gallus_gallus/Gene/Summary?db=core;g=ENSGALG00010000784) | [FBXL3](http://www.ensembl.org/homo_sapiens/Gene/Summary?g=ENSG00000005812) | [FBXL3](http://www.ensembl.org/gallus_gallus/Gene/Summary?db=core;g=ENSGALG00010000784) |
| [ENSGALG00010001127](http://www.ensembl.org/gallus_gallus/Gene/Summary?db=core;g=ENSGALG00010001127) | [SIAH2](http://www.ensembl.org/homo_sapiens/Gene/Summary?g=ENSG00000181788) | [SIAH2](http://www.ensembl.org/gallus_gallus/Gene/Summary?db=core;g=ENSGALG00010001127) |
| [ENSGALG00010001510](http://www.ensembl.org/gallus_gallus/Gene/Summary?db=core;g=ENSGALG00010001510) | [AP1S2](http://www.ensembl.org/homo_sapiens/Gene/Summary?g=ENSG00000182287) | [AP1S2](http://www.ensembl.org/gallus_gallus/Gene/Summary?db=core;g=ENSGALG00010001510) |
| [ENSGALG00010002102](http://www.ensembl.org/gallus_gallus/Gene/Summary?db=core;g=ENSGALG00010002102) | [PAK1](http://www.ensembl.org/homo_sapiens/Gene/Summary?g=ENSG00000149269) | [PAK1](http://www.ensembl.org/gallus_gallus/Gene/Summary?db=core;g=ENSGALG00010002102) |
| [ENSGALG00010003110](http://www.ensembl.org/gallus_gallus/Gene/Summary?db=core;g=ENSGALG00010003110) | - | [BF1](http://www.ensembl.org/gallus_gallus/Gene/Summary?db=core;g=ENSGALG00010003110) |
| [ENSGALG00010003229](http://www.ensembl.org/gallus_gallus/Gene/Summary?db=core;g=ENSGALG00010003229) | [STIM1](http://www.ensembl.org/homo_sapiens/Gene/Summary?g=ENSG00000167323) | [STIM1](http://www.ensembl.org/gallus_gallus/Gene/Summary?db=core;g=ENSGALG00010003229) |
| [ENSGALG00010003383](http://www.ensembl.org/gallus_gallus/Gene/Summary?db=core;g=ENSGALG00010003383) | [UBE2J1](http://www.ensembl.org/homo_sapiens/Gene/Summary?g=ENSG00000198833) | [UBE2J1](http://www.ensembl.org/gallus_gallus/Gene/Summary?db=core;g=ENSGALG00010003383) |
| [ENSGALG00010003874](http://www.ensembl.org/gallus_gallus/Gene/Summary?db=core;g=ENSGALG00010003874) | [YES1](http://www.ensembl.org/homo_sapiens/Gene/Summary?g=ENSG00000176105) | [YES1](http://www.ensembl.org/gallus_gallus/Gene/Summary?db=core;g=ENSGALG00010003874) |
| [ENSGALG00010004044](http://www.ensembl.org/gallus_gallus/Gene/Summary?db=core;g=ENSGALG00010004044) | [RNF138](http://www.ensembl.org/homo_sapiens/Gene/Summary?g=ENSG00000134758) | [RNF138](http://www.ensembl.org/gallus_gallus/Gene/Summary?db=core;g=ENSGALG00010004044) |
| [ENSGALG00010005166](http://www.ensembl.org/gallus_gallus/Gene/Summary?db=core;g=ENSGALG00010005166) | [CBLB](http://www.ensembl.org/homo_sapiens/Gene/Summary?g=ENSG00000114423) | [CBLB](http://www.ensembl.org/gallus_gallus/Gene/Summary?db=core;g=ENSGALG00010005166) |
| [ENSGALG00010005981](http://www.ensembl.org/gallus_gallus/Gene/Summary?db=core;g=ENSGALG00010005981) | [AREL1](http://www.ensembl.org/homo_sapiens/Gene/Summary?g=ENSG00000119682) | [AREL1](http://www.ensembl.org/gallus_gallus/Gene/Summary?db=core;g=ENSGALG00010005981) |
| [ENSGALG00010006343](http://www.ensembl.org/gallus_gallus/Gene/Summary?db=core;g=ENSGALG00010006343) | [SEC23A](http://www.ensembl.org/homo_sapiens/Gene/Summary?g=ENSG00000100934) | [SEC23A](http://www.ensembl.org/gallus_gallus/Gene/Summary?db=core;g=ENSGALG00010006343) |
| [ENSGALG00010006838](http://www.ensembl.org/gallus_gallus/Gene/Summary?db=core;g=ENSGALG00010006838) | [ASB13](http://www.ensembl.org/homo_sapiens/Gene/Summary?g=ENSG00000196372) | [ASB13](http://www.ensembl.org/gallus_gallus/Gene/Summary?db=core;g=ENSGALG00010006838) |
| [ENSGALG00010007849](http://www.ensembl.org/gallus_gallus/Gene/Summary?db=core;g=ENSGALG00010007849) | [CXADR](http://www.ensembl.org/homo_sapiens/Gene/Summary?g=ENSG00000154639) | [CXADR](http://www.ensembl.org/gallus_gallus/Gene/Summary?db=core;g=ENSGALG00010007849) |
| [ENSGALG00010008300](http://www.ensembl.org/gallus_gallus/Gene/Summary?db=core;g=ENSGALG00010008300) | [CUL1](http://www.ensembl.org/homo_sapiens/Gene/Summary?g=ENSG00000055130) | [CUL1](http://www.ensembl.org/gallus_gallus/Gene/Summary?db=core;g=ENSGALG00010008300) |
| [ENSGALG00010008426](http://www.ensembl.org/gallus_gallus/Gene/Summary?db=core;g=ENSGALG00010008426) | [AP1G1](http://www.ensembl.org/homo_sapiens/Gene/Summary?g=ENSG00000166747) | [AP1G1](http://www.ensembl.org/gallus_gallus/Gene/Summary?db=core;g=ENSGALG00010008426) |
| [ENSGALG00010008431](http://www.ensembl.org/gallus_gallus/Gene/Summary?db=core;g=ENSGALG00010008431) | [SH3RF1](http://www.ensembl.org/homo_sapiens/Gene/Summary?g=ENSG00000154447) | [SH3RF1](http://www.ensembl.org/gallus_gallus/Gene/Summary?db=core;g=ENSGALG00010008431) |
| [ENSGALG00010009340](http://www.ensembl.org/gallus_gallus/Gene/Summary?db=core;g=ENSGALG00010009340) | [SYK](http://www.ensembl.org/homo_sapiens/Gene/Summary?g=ENSG00000165025) | [SYK](http://www.ensembl.org/gallus_gallus/Gene/Summary?db=core;g=ENSGALG00010009340) |
| [ENSGALG00010010352](http://www.ensembl.org/gallus_gallus/Gene/Summary?db=core;g=ENSGALG00010010352) | [MALT1](http://www.ensembl.org/homo_sapiens/Gene/Summary?g=ENSG00000172175) | [MALT1](http://www.ensembl.org/gallus_gallus/Gene/Summary?db=core;g=ENSGALG00010010352) |
| [ENSGALG00010010743](http://www.ensembl.org/gallus_gallus/Gene/Summary?db=core;g=ENSGALG00010010743) | [KLHL20](http://www.ensembl.org/homo_sapiens/Gene/Summary?g=ENSG00000076321) | [KLHL20](http://www.ensembl.org/gallus_gallus/Gene/Summary?db=core;g=ENSGALG00010010743) |
| [ENSGALG00010011406](http://www.ensembl.org/gallus_gallus/Gene/Summary?db=core;g=ENSGALG00010011406) | [UBE2R2](http://www.ensembl.org/homo_sapiens/Gene/Summary?g=ENSG00000107341) | [UBE2R2](http://www.ensembl.org/gallus_gallus/Gene/Summary?db=core;g=ENSGALG00010011406) |
| [ENSGALG00010012419](http://www.ensembl.org/gallus_gallus/Gene/Summary?db=core;g=ENSGALG00010012419) | [HACE1](http://www.ensembl.org/homo_sapiens/Gene/Summary?g=ENSG00000085382) | [HACE1](http://www.ensembl.org/gallus_gallus/Gene/Summary?db=core;g=ENSGALG00010012419) |
| [ENSGALG00010012859](http://www.ensembl.org/gallus_gallus/Gene/Summary?db=core;g=ENSGALG00010012859) | - | [FBXL21](http://www.ensembl.org/gallus_gallus/Gene/Summary?db=core;g=ENSGALG00010012859) |
| [ENSGALG00010012997](http://www.ensembl.org/gallus_gallus/Gene/Summary?db=core;g=ENSGALG00010012997) | [UBE2A](http://www.ensembl.org/homo_sapiens/Gene/Summary?g=ENSG00000077721) | [UBE2A](http://www.ensembl.org/gallus_gallus/Gene/Summary?db=core;g=ENSGALG00010012997) |
| [ENSGALG00010013241](http://www.ensembl.org/gallus_gallus/Gene/Summary?db=core;g=ENSGALG00010013241) | [SEC24A](http://www.ensembl.org/homo_sapiens/Gene/Summary?g=ENSG00000113615) | [SEC24A](http://www.ensembl.org/gallus_gallus/Gene/Summary?db=core;g=ENSGALG00010013241) |
| [ENSGALG00010013246](http://www.ensembl.org/gallus_gallus/Gene/Summary?db=core;g=ENSGALG00010013246) | [LNPEP](http://www.ensembl.org/homo_sapiens/Gene/Summary?g=ENSG00000113441) | [LNPEP](http://www.ensembl.org/gallus_gallus/Gene/Summary?db=core;g=ENSGALG00010013246) |
| [ENSGALG00010013354](http://www.ensembl.org/gallus_gallus/Gene/Summary?db=core;g=ENSGALG00010013354) | [WWP1](http://www.ensembl.org/homo_sapiens/Gene/Summary?g=ENSG00000123124) | [WWP1](http://www.ensembl.org/gallus_gallus/Gene/Summary?db=core;g=ENSGALG00010013354) |
| [ENSGALG00010013357](http://www.ensembl.org/gallus_gallus/Gene/Summary?db=core;g=ENSGALG00010013357) | [RICTOR](http://www.ensembl.org/homo_sapiens/Gene/Summary?g=ENSG00000164327) | [RICTOR](http://www.ensembl.org/gallus_gallus/Gene/Summary?db=core;g=ENSGALG00010013357) |
| [ENSGALG00010013396](http://www.ensembl.org/gallus_gallus/Gene/Summary?db=core;g=ENSGALG00010013396) | [FYB1](http://www.ensembl.org/homo_sapiens/Gene/Summary?g=ENSG00000082074) | [FYB1](http://www.ensembl.org/gallus_gallus/Gene/Summary?db=core;g=ENSGALG00010013396) |
| [ENSGALG00010013408](http://www.ensembl.org/gallus_gallus/Gene/Summary?db=core;g=ENSGALG00010013408) | [RLIM](http://www.ensembl.org/homo_sapiens/Gene/Summary?g=ENSG00000131263) | [RLIM](http://www.ensembl.org/gallus_gallus/Gene/Summary?db=core;g=ENSGALG00010013408) |
| [ENSGALG00010014143](http://www.ensembl.org/gallus_gallus/Gene/Summary?db=core;g=ENSGALG00010014143) | [NFATC2](http://www.ensembl.org/homo_sapiens/Gene/Summary?g=ENSG00000101096) | [NFATC2](http://www.ensembl.org/gallus_gallus/Gene/Summary?db=core;g=ENSGALG00010014143) |
| [ENSGALG00010014494](http://www.ensembl.org/gallus_gallus/Gene/Summary?db=core;g=ENSGALG00010014494) | [KLHL9](http://www.ensembl.org/homo_sapiens/Gene/Summary?g=ENSG00000198642) | [KLHL9](http://www.ensembl.org/gallus_gallus/Gene/Summary?db=core;g=ENSGALG00010014494) |
| [ENSGALG00010015443](http://www.ensembl.org/gallus_gallus/Gene/Summary?db=core;g=ENSGALG00010015443) | [PAK3](http://www.ensembl.org/homo_sapiens/Gene/Summary?g=ENSG00000077264) | [PAK3](http://www.ensembl.org/gallus_gallus/Gene/Summary?db=core;g=ENSGALG00010015443) |
| [ENSGALG00010015520](http://www.ensembl.org/gallus_gallus/Gene/Summary?db=core;g=ENSGALG00010015520) | [UBE3A](http://www.ensembl.org/homo_sapiens/Gene/Summary?g=ENSG00000114062) | [UBE3A](http://www.ensembl.org/gallus_gallus/Gene/Summary?db=core;g=ENSGALG00010015520) |
| [ENSGALG00010015788](http://www.ensembl.org/gallus_gallus/Gene/Summary?db=core;g=ENSGALG00010015788) | [TRPC1](http://www.ensembl.org/homo_sapiens/Gene/Summary?g=ENSG00000144935) | [TRPC1](http://www.ensembl.org/gallus_gallus/Gene/Summary?db=core;g=ENSGALG00010015788) |
| [ENSGALG00010015861](http://www.ensembl.org/gallus_gallus/Gene/Summary?db=core;g=ENSGALG00010015861) | [PPP2CA](http://www.ensembl.org/homo_sapiens/Gene/Summary?g=ENSG00000113575) | [PPP2CA](http://www.ensembl.org/gallus_gallus/Gene/Summary?db=core;g=ENSGALG00010015861) |
| [ENSGALG00010016295](http://www.ensembl.org/gallus_gallus/Gene/Summary?db=core;g=ENSGALG00010016295) | [UBE2K](http://www.ensembl.org/homo_sapiens/Gene/Summary?g=ENSG00000078140) | [UBE2K](http://www.ensembl.org/gallus_gallus/Gene/Summary?db=core;g=ENSGALG00010016295) |
| [ENSGALG00010017035](http://www.ensembl.org/gallus_gallus/Gene/Summary?db=core;g=ENSGALG00010017035) | [PIK3CD](http://www.ensembl.org/homo_sapiens/Gene/Summary?g=ENSG00000171608) | [PIK3CD](http://www.ensembl.org/gallus_gallus/Gene/Summary?db=core;g=ENSGALG00010017035) |
| [ENSGALG00010017119](http://www.ensembl.org/gallus_gallus/Gene/Summary?db=core;g=ENSGALG00010017119) | [BTBD6](http://www.ensembl.org/homo_sapiens/Gene/Summary?g=ENSG00000184887) | [BTBD6](http://www.ensembl.org/gallus_gallus/Gene/Summary?db=core;g=ENSGALG00010017119) |
| [ENSGALG00010017280](http://www.ensembl.org/gallus_gallus/Gene/Summary?db=core;g=ENSGALG00010017280) | [SOS1](http://www.ensembl.org/homo_sapiens/Gene/Summary?g=ENSG00000115904) | [SOS1](http://www.ensembl.org/gallus_gallus/Gene/Summary?db=core;g=ENSGALG00010017280) |
| [ENSGALG00010017364](http://www.ensembl.org/gallus_gallus/Gene/Summary?db=core;g=ENSGALG00010017364) | [AKT1](http://www.ensembl.org/homo_sapiens/Gene/Summary?g=ENSG00000142208) | [AKT1](http://www.ensembl.org/gallus_gallus/Gene/Summary?db=core;g=ENSGALG00010017364) |
| [ENSGALG00010017609](http://www.ensembl.org/gallus_gallus/Gene/Summary?db=core;g=ENSGALG00010017609) | [TRIM71](http://www.ensembl.org/homo_sapiens/Gene/Summary?g=ENSG00000206557) | [TRIM71](http://www.ensembl.org/gallus_gallus/Gene/Summary?db=core;g=ENSGALG00010017609) |
| [ENSGALG00010018355](http://www.ensembl.org/gallus_gallus/Gene/Summary?db=core;g=ENSGALG00010018355) | [PRKCB](http://www.ensembl.org/homo_sapiens/Gene/Summary?g=ENSG00000166501) | [PRKCB](http://www.ensembl.org/gallus_gallus/Gene/Summary?db=core;g=ENSGALG00010018355) |
| [ENSGALG00010019023](http://www.ensembl.org/gallus_gallus/Gene/Summary?db=core;g=ENSGALG00010019023) | [AP2M1](http://www.ensembl.org/homo_sapiens/Gene/Summary?g=ENSG00000161203) | [AP2M1](http://www.ensembl.org/gallus_gallus/Gene/Summary?db=core;g=ENSGALG00010019023) |
| [ENSGALG00010019036](http://www.ensembl.org/gallus_gallus/Gene/Summary?db=core;g=ENSGALG00010019036) | [REL](http://www.ensembl.org/homo_sapiens/Gene/Summary?g=ENSG00000162924) | [REL](http://www.ensembl.org/gallus_gallus/Gene/Summary?db=core;g=ENSGALG00010019036) |
| [ENSGALG00010019529](http://www.ensembl.org/gallus_gallus/Gene/Summary?db=core;g=ENSGALG00010019529) | [SNAP23](http://www.ensembl.org/homo_sapiens/Gene/Summary?g=ENSG00000092531) | [SNAP23](http://www.ensembl.org/gallus_gallus/Gene/Summary?db=core;g=ENSGALG00010019529) |
| [ENSGALG00010019650](http://www.ensembl.org/gallus_gallus/Gene/Summary?db=core;g=ENSGALG00010019650) | [KBTBD8](http://www.ensembl.org/homo_sapiens/Gene/Summary?g=ENSG00000163376) | [KBTBD8](http://www.ensembl.org/gallus_gallus/Gene/Summary?db=core;g=ENSGALG00010019650) |
| [ENSGALG00010020199](http://www.ensembl.org/gallus_gallus/Gene/Summary?db=core;g=ENSGALG00010020199) | [DET1](http://www.ensembl.org/homo_sapiens/Gene/Summary?g=ENSG00000140543) | [DET1](http://www.ensembl.org/gallus_gallus/Gene/Summary?db=core;g=ENSGALG00010020199) |
| [ENSGALG00010020390](http://www.ensembl.org/gallus_gallus/Gene/Summary?db=core;g=ENSGALG00010020390) | [KLHL25](http://www.ensembl.org/homo_sapiens/Gene/Summary?g=ENSG00000183655) | [KLHL25](http://www.ensembl.org/gallus_gallus/Gene/Summary?db=core;g=ENSGALG00010020390) |
| [ENSGALG00010020701](http://www.ensembl.org/gallus_gallus/Gene/Summary?db=core;g=ENSGALG00010020701) | [PTPN11](http://www.ensembl.org/homo_sapiens/Gene/Summary?g=ENSG00000179295) | [PTPN11](http://www.ensembl.org/gallus_gallus/Gene/Summary?db=core;g=ENSGALG00010020701) |
| [ENSGALG00010020732](http://www.ensembl.org/gallus_gallus/Gene/Summary?db=core;g=ENSGALG00010020732) | [ARIH2](http://www.ensembl.org/homo_sapiens/Gene/Summary?g=ENSG00000177479) | [ARIH2](http://www.ensembl.org/gallus_gallus/Gene/Summary?db=core;g=ENSGALG00010020732) |
| [ENSGALG00010021210](http://www.ensembl.org/gallus_gallus/Gene/Summary?db=core;g=ENSGALG00010021210) | - | SEC24C |
| [ENSGALG00010021217](http://www.ensembl.org/gallus_gallus/Gene/Summary?db=core;g=ENSGALG00010021217) | [FBXO2](http://www.ensembl.org/homo_sapiens/Gene/Summary?g=ENSG00000116661) | [FBXO2](http://www.ensembl.org/gallus_gallus/Gene/Summary?db=core;g=ENSGALG00010021217) |
| [ENSGALG00010021335](http://www.ensembl.org/gallus_gallus/Gene/Summary?db=core;g=ENSGALG00010021335) | [NFATC3](http://www.ensembl.org/homo_sapiens/Gene/Summary?g=ENSG00000072736) | [NFATC3](http://www.ensembl.org/gallus_gallus/Gene/Summary?db=core;g=ENSGALG00010021335) |
| [ENSGALG00010021746](http://www.ensembl.org/gallus_gallus/Gene/Summary?db=core;g=ENSGALG00010021746) | [MTOR](http://www.ensembl.org/homo_sapiens/Gene/Summary?g=ENSG00000198793) | [MTOR](http://www.ensembl.org/gallus_gallus/Gene/Summary?db=core;g=ENSGALG00010021746) |
| [ENSGALG00010021807](http://www.ensembl.org/gallus_gallus/Gene/Summary?db=core;g=ENSGALG00010021807) | [CDC42](http://www.ensembl.org/homo_sapiens/Gene/Summary?g=ENSG00000070831) | [CDC42](http://www.ensembl.org/gallus_gallus/Gene/Summary?db=core;g=ENSGALG00010021807) |
| [ENSGALG00010022076](http://www.ensembl.org/gallus_gallus/Gene/Summary?db=core;g=ENSGALG00010022076) | [YWHAB](http://www.ensembl.org/homo_sapiens/Gene/Summary?g=ENSG00000166913) | [YWHAB](http://www.ensembl.org/gallus_gallus/Gene/Summary?db=core;g=ENSGALG00010022076) |
| [ENSGALG00010024250](http://www.ensembl.org/gallus_gallus/Gene/Summary?db=core;g=ENSGALG00010024250) | [AP2A2](http://www.ensembl.org/homo_sapiens/Gene/Summary?g=ENSG00000183020) | [AP2A2](http://www.ensembl.org/gallus_gallus/Gene/Summary?db=core;g=ENSGALG00010024250) |
| [ENSGALG00010024624](http://www.ensembl.org/gallus_gallus/Gene/Summary?db=core;g=ENSGALG00010024624) | [CTSD](http://www.ensembl.org/homo_sapiens/Gene/Summary?g=ENSG00000117984) | [CTSD](http://www.ensembl.org/gallus_gallus/Gene/Summary?db=core;g=ENSGALG00010024624) |
| [ENSGALG00010024699](http://www.ensembl.org/gallus_gallus/Gene/Summary?db=core;g=ENSGALG00010024699) | [FBXL8](http://www.ensembl.org/homo_sapiens/Gene/Summary?g=ENSG00000135722) | [FBXL8](http://www.ensembl.org/gallus_gallus/Gene/Summary?db=core;g=ENSGALG00010024699) |
| [ENSGALG00010024742](http://www.ensembl.org/gallus_gallus/Gene/Summary?db=core;g=ENSGALG00010024742) | [PAK2](http://www.ensembl.org/homo_sapiens/Gene/Summary?g=ENSG00000180370) | [PAK2](http://www.ensembl.org/gallus_gallus/Gene/Summary?db=core;g=ENSGALG00010024742) |
| [ENSGALG00010025125](http://www.ensembl.org/gallus_gallus/Gene/Summary?db=core;g=ENSGALG00010025125) | [PIK3CB](http://www.ensembl.org/homo_sapiens/Gene/Summary?g=ENSG00000051382) | [PIK3CB](http://www.ensembl.org/gallus_gallus/Gene/Summary?db=core;g=ENSGALG00010025125) |
| [ENSGALG00010025204](http://www.ensembl.org/gallus_gallus/Gene/Summary?db=core;g=ENSGALG00010025204) | [TRAF6](http://www.ensembl.org/homo_sapiens/Gene/Summary?g=ENSG00000175104) | [TRAF6](http://www.ensembl.org/gallus_gallus/Gene/Summary?db=core;g=ENSGALG00010025204) |
| [ENSGALG00010025245](http://www.ensembl.org/gallus_gallus/Gene/Summary?db=core;g=ENSGALG00010025245) | - | RAP1A |
| [ENSGALG00010025512](http://www.ensembl.org/gallus_gallus/Gene/Summary?db=core;g=ENSGALG00010025512) | [NRAS](http://www.ensembl.org/homo_sapiens/Gene/Summary?g=ENSG00000213281) | [NRAS](http://www.ensembl.org/gallus_gallus/Gene/Summary?db=core;g=ENSGALG00010025512) |
| [ENSGALG00010025590](http://www.ensembl.org/gallus_gallus/Gene/Summary?db=core;g=ENSGALG00010025590) | [HRAS](http://www.ensembl.org/homo_sapiens/Gene/Summary?g=ENSG00000174775) | [HRAS](http://www.ensembl.org/gallus_gallus/Gene/Summary?db=core;g=ENSGALG00010025590) |
| [ENSGALG00010026097](http://www.ensembl.org/gallus_gallus/Gene/Summary?db=core;g=ENSGALG00010026097) | [ARF1](http://www.ensembl.org/homo_sapiens/Gene/Summary?g=ENSG00000143761) | [ARF1](http://www.ensembl.org/gallus_gallus/Gene/Summary?db=core;g=ENSGALG00010026097) |
| [ENSGALG00010026237](http://www.ensembl.org/gallus_gallus/Gene/Summary?db=core;g=ENSGALG00010026237) | [KLHL22](http://www.ensembl.org/homo_sapiens/Gene/Summary?g=ENSG00000099910) | [KLHL22](http://www.ensembl.org/gallus_gallus/Gene/Summary?db=core;g=ENSGALG00010026237) |
| [ENSGALG00010027324](http://www.ensembl.org/gallus_gallus/Gene/Summary?db=core;g=ENSGALG00010027324) | [PIK3R2](http://www.ensembl.org/homo_sapiens/Gene/Summary?g=ENSG00000105647) | [PIK3R2](http://www.ensembl.org/gallus_gallus/Gene/Summary?db=core;g=ENSGALG00010027324) |
| [ENSGALG00010027930](http://www.ensembl.org/gallus_gallus/Gene/Summary?db=core;g=ENSGALG00010027930) | [RAF1](http://www.ensembl.org/homo_sapiens/Gene/Summary?g=ENSG00000132155) | [RAF1](http://www.ensembl.org/gallus_gallus/Gene/Summary?db=core;g=ENSGALG00010027930) |
| [ENSGALG00010027946](http://www.ensembl.org/gallus_gallus/Gene/Summary?db=core;g=ENSGALG00010027946) | [AP1M1](http://www.ensembl.org/homo_sapiens/Gene/Summary?g=ENSG00000072958) | [AP1M1](http://www.ensembl.org/gallus_gallus/Gene/Summary?db=core;g=ENSGALG00010027946) |
| [ENSGALG00010028139](http://www.ensembl.org/gallus_gallus/Gene/Summary?db=core;g=ENSGALG00010028139) | [FZR1](http://www.ensembl.org/homo_sapiens/Gene/Summary?g=ENSG00000105325) | [FZR1](http://www.ensembl.org/gallus_gallus/Gene/Summary?db=core;g=ENSGALG00010028139) |
| [ENSGALG00010028473](http://www.ensembl.org/gallus_gallus/Gene/Summary?db=core;g=ENSGALG00010028473) | [CDC34](http://www.ensembl.org/homo_sapiens/Gene/Summary?g=ENSG00000099804) | [CDC34](http://www.ensembl.org/gallus_gallus/Gene/Summary?db=core;g=ENSGALG00010028473) |
| [ENSGALG00010029429](http://www.ensembl.org/gallus_gallus/Gene/Summary?db=core;g=ENSGALG00010029429) | [UBE2G1](http://www.ensembl.org/homo_sapiens/Gene/Summary?g=ENSG00000132388) | [UBE2G1](http://www.ensembl.org/gallus_gallus/Gene/Summary?db=core;g=ENSGALG00010029429) |
| [ENSGALG00010029661](http://www.ensembl.org/gallus_gallus/Gene/Summary?db=core;g=ENSGALG00010029661) | [CD300C](http://www.ensembl.org/homo_sapiens/Gene/Summary?g=ENSG00000167850) | [KRABZFP](http://www.ensembl.org/gallus_gallus/Gene/Summary?db=core;g=ENSGALG00010029661) |
| [ENSGALG00010029705](http://www.ensembl.org/gallus_gallus/Gene/Summary?db=core;g=ENSGALG00010029705) | [GRB2](http://www.ensembl.org/homo_sapiens/Gene/Summary?g=ENSG00000177885) | [GRB2](http://www.ensembl.org/gallus_gallus/Gene/Summary?db=core;g=ENSGALG00010029705) |
| Last accession Biomart (Ensembl): May 08, 2025 | | |

**Table S3:** Functional terms identified and their interconnections in jejunum at 7d for by Cluego – Cytoscape identified in GSEA analysis

| ID | Term | Term P-Value* | Group P-Value * | Associated Genes % | Associated Genes |
| --- | --- | --- | --- | --- | --- |
| GO:0002702 | Positive regulation of production of molecular mediator of immune response | 0.21 | 0.01 | 4.41 | PCASP1, SYK, TRAF6 |
| GO:0002705 | Positive regulation of leukocyte mediated immunity | 0.33 | 0.01 | 6.45 | AP1G1, KLHL22, PCASP1, TRAF6 |
| GO:0002718 | Regulation of cytokine production involved in immune response | 0.33 | 0.01 | 6.45 | PCASP1, SYK, TRAF6, UBE2J1 |
| GO:0002821 | Positive regulation of adaptive immune response | 0.32 | 0.01 | 4.92 | KLHL22, PCASP1, TRAF6 |
| GO:0002456 | T cell mediated immunity | 0.25 | 0.01 | 4.84 | KLHL22, PCASP1, TRAF6 |
| GO:0002706 | Regulation of lymphocyte mediated immunity | 0.19 | 0.01 | 6.67 | AP1G1, GRB2, KLHL22, PCASP1, TRAF6 |
| GO:0002720 | Positive regulation of cytokine production involved in immune response | 0.41 | 0.01 | 6.98 | PCASP1, SYK, TRAF6 |
| GO:0002768 | Immune response-regulating cell surface receptor signaling pathway | 0.05 | 0.01 | 5.06 | CBLB, GRB2, HRAS, NFATC2, PCASP1, PRKCB, SOS1, SYK, TRAF6 |
| GO:0042088 | T-helper 1 type immune response | 0.10 | 0.01 | 15.79 | HRAS, MTOR, TRAF6 |
| GO:0042098 | T cell proliferation | 0.22 | 0.01 | 5.36 | CBLB, PCASP1, PTPN11, SOS1, SYK, TRAF6 |
| GO:0046631 | Alpha-beta T cell activation | 0.41 | 0.01 | 4.72 | CBLB, KLHL25, MTOR, PCASP1, SYK |
| GO:0050863 | Regulation of T cell activation | 0.25 | 0.01 | 4.19 | CBLB, KLHL22, KLHL25, PCASP1, PTPN11, SOS1, SYK, TRAF6 |
| GO:0002708 | Positive regulation of lymphocyte mediated immunity | 0.29 | 0.01 | 7.14 | AP1G1, KLHL22, PCASP1, TRAF6 |
| GO:0002824 | Positive regulation of adaptive immune response based on somatic recombination of immune receptors built from immunoglobulin superfamily domains | 0.33 | 0.01 | 5.36 | KLHL22, PCASP1, TRAF6 |
| GO:0045621 | Positive regulation of lymphocyte differentiation | 0.21 | 0.01 | 4.41 | KLHL25, PCASP1, SYK |
| GO:0050671 | Positive regulation of lymphocyte proliferation | 0.12 | 0.01 | 4.11 | NFATC2, SYK, TRAF6 |
| GO:0050870 | Positive regulation of T cell activation | 0.48 | 0.01 | 4.00 | KLHL22, KLHL25, PCASP1, SYK, TRAF6 |
| **GO:0002429** | **Immune response-activating cell surface receptor signaling pathway** | **0.04** | **0.01** | **5.20** | **CBLB, GRB2, HRAS, NFATC2, PCASP1, PRKCB, SOS1, SYK, TRAF6** |
| GO:0002709 | Regulation of T cell mediated immunity | 0.39 | 0.01 | 8.11 | KLHL22, PCASP1, TRAF6 |
| GO:0035710 | CD4-positive, alpha-beta T cell activation | 0.38 | 0.01 | 5.88 | CBLB, KLHL25, MTOR, PCASP1 |
| GO:0045580 | Regulation of T cell differentiation | 0.41 | 0.01 | 4.49 | KLHL25, PCASP1, SOS1, SYK |
| GO:0046632 | Alpha-beta T cell differentiation | 0.40 | 0.01 | 5.26 | KLHL25, MTOR, PCASP1, SYK |
| GO:0046634 | Regulation of alpha-beta T cell activation | 0.38 | 0.01 | 5.97 | CBLB, KLHL25, PCASP1, SYK |
| GO:0002711 | Positive regulation of T cell mediated immunity | 0.27 | 0.01 | 10.34 | KLHL22, PCASP1, TRAF6 |
| GO:0045582 | Positive regulation of T cell differentiation | 0.33 | 0.01 | 5.36 | KLHL25, PCASP1, SYK |
| GO:0046635 | Positive regulation of alpha-beta T cell activation | 0.43 | 0.01 | 6.38 | KLHL25, PCASP1, SYK |
| GO:0050851 | Antigen receptor-mediated signaling pathway | 0.00 | 0.01 | 7.56 | CBLB, GRB2, HRAS, NFATC2, PCASP1, PRKCB, SOS1, SYK, TRAF6 |
| GO:0043367 | CD4-positive, alpha-beta T cell differentiation | 0.46 | 0.01 | 5.66 | KLHL25, MTOR, PCASP1 |
| GO:0046637 | Regulation of alpha-beta T cell differentiation | 0.41 | 0.01 | 6.98 | KLHL25, PCASP1, SYK |
| GO:2000514 | Regulation of CD4-positive, alpha-beta T cell activation | 0.41 | 0.01 | 7.14 | CBLB, KLHL25, PCASP1 |
| GO:0046638 | Positive regulation of alpha-beta T cell differentiation | 0.32 | 0.01 | 9.09 | KLHL25, PCASP1, SYK |
| GO:0050852 | T cell receptor signaling pathway | 0.38 | 0.01 | 4.40 | CBLB, HRAS, PCASP1, TRAF6 |
| GO:0002702 | Positive regulation of production of molecular mediator of immune response | 0.21 | 0.03 | 4.41 | PCASP1, SYK, TRAF6 |
| GO:0002718 | Regulation of cytokine production involved in immune response | 0.33 | 0.03 | 6.45 | PCASP1, SYK, TRAF6, UBE2J1 |
| GO:0002720 | Positive regulation of cytokine production involved in immune response | 0.41 | 0.03 | 6.98 | PCASP1, SYK, TRAF6 |
| GO:0042098 | T cell proliferation | 0.22 | 0.03 | 5.36 | CBLB, PCASP1, PTPN11, SOS1, SYK, TRAF6 |
| GO:0050670 | Regulation of lymphocyte proliferation | 0.29 | 0.03 | 4.88 | CBLB, NFATC2, PTPN11, SOS1, SYK, TRAF6 |
| GO:0050863 | Regulation of T cell activation | 0.25 | 0.03 | 4.19 | CBLB, KLHL22, KLHL25, PCASP1, PTPN11, SOS1, SYK, TRAF6 |
| **GO:0050671** | **Positive regulation of lymphocyte proliferation** | **0.12** | **0.03** | **4.11** | **NFATC2, SYK, TRAF6** |
| GO:0050870 | Positive regulation of T cell activation | 0.48 | 0.03 | 4.00 | KLHL22, KLHL25, PCASP1, SYK, TRAF6 |
| GO:0042129 | Regulation of T cell proliferation | 0.32 | 0.03 | 5.49 | CBLB, PTPN11, SOS1, SYK, TRAF6 |
| GO:0045580 | Regulation of T cell differentiation | 0.41 | 0.03 | 4.49 | KLHL25, PCASP1, SOS1, SYK |
| GO:0046634 | Regulation of alpha-beta T cell activation | 0.38 | 0.03 | 5.97 | CBLB, KLHL25, PCASP1, SYK |
| GO:0050852 | T cell receptor signaling pathway | 0.38 | 0.03 | 4.40 | CBLB, HRAS, PCASP1, TRAF6 |
| GO:0002768 | Immune response-regulating cell surface receptor signaling pathway | 0.05 | 0.01 | 5.06 | CBLB, GRB2, HRAS, NFATC2, PCASP1, PRKCB, SOS1, SYK, TRAF6 |
| GO:0002429 | Immune response-activating cell surface receptor signaling pathway | 0.04 | 0.01 | 5.20 | CBLB, GRB2, HRAS, NFATC2, PCASP1, PRKCB, SOS1, SYK, TRAF6 |
| **GO:0050851** | **Antigen receptor-mediated signaling pathway** | **0.00** | **0.01** | **7.56** | **CBLB, GRB2, HRAS, NFATC2, PCASP1, PRKCB, SOS1, SYK, TRAF6** |
| GO:0050852 | T cell receptor signaling pathway | 0.38 | 0.01 | 4.40 | CBLB, HRAS, PCASP1, TRAF6 |
| GO:0050853 | B cell receptor signaling pathway | 0.02 | 0.01 | 11.90 | GRB2, NFATC2, PRKCB, SOS1, SYK |
| GO:0002228 | Natural killer cell mediated immunity | 0.27 | 0.02 | 10.34 | AP1G1, GRB2, PIK3CB |
| **GO:0042267** | **Natural killer cell mediated cytotoxicity** | **0.22** | **0.02** | **11.54** | **AP1G1, GRB2, PIK3CB** |

GO_ImmuneSystemProcess-EBI-UniProt-GOA-ACAP-ARAP

Analysis performed with 75 genes (Gallus gallus name) in ClueGo – Cytoscape software

*Corrected with Bonferroni
